# Deubiquitinase USP2a Sustains Interferons Antiviral Activity by Restricting Ubiquitination of Activated STAT1 in the Nucleus

**DOI:** 10.1371/journal.ppat.1005764

**Published:** 2016-07-19

**Authors:** Ying Ren, Peng Zhao, Jin Liu, Yukang Yuan, Qiao Cheng, Yibo Zuo, Liping Qian, Chang Liu, Tingting Guo, Liting Zhang, Xiaofang Wang, Guanghui Qian, Lemin Li, Jun Ge, Jianfeng Dai, Sidong Xiong, Hui Zheng

**Affiliations:** 1 Institutes of Biology and Medical Sciences, Soochow University, Suzhou, China; 2 Jiangsu Key Laboratory of Infection and Immunity, Soochow University, Jiangsu, China; University of Pittsburgh, UNITED STATES

## Abstract

STAT1 is a critical transcription factor for regulating host antiviral defenses. STAT1 activation is largely dependent on phosphorylation at tyrosine 701 site of STAT1 (pY701-STAT1). Understanding how pY701-STAT1 is regulated by intracellular signaling remains a major challenge. Here we find that pY701-STAT1 is the major form of ubiquitinated-STAT1 induced by interferons (IFNs). While total STAT1 remains relatively stable during the early stages of IFNs signaling, pY701-STAT1 can be rapidly downregulated by the ubiquitin-proteasome system. Moreover, ubiquitinated pY701-STAT1 is located predominantly in the nucleus, and inhibiting nuclear import of pY701-STAT1 significantly blocks ubiquitination and downregulation of pY701-STAT1. Furthermore, we reveal that the deubiquitinase USP2a translocates into the nucleus and binds to pY701-STAT1, and inhibits K48-linked ubiquitination and degradation of pY701-STAT1. Importantly, USP2a sustains IFNs-induced pY701-STAT1 levels, and enhances all three classes of IFNs- mediated signaling and antiviral activity. To our knowledge, this is the first identified deubiquitinase that targets activated pY701-STAT1. These findings uncover a positive mechanism by which IFNs execute efficient antiviral signaling and function, and may provide potential targets for improving IFNs-based antiviral therapy.

## Author Summary

Phosphorylated STAT1 at tyrosine 701 site (pY701-STAT1) is critical for regulating many cellular functions including antiviral immunity. Maintaining sufficient pY701-STAT1 levels in the nucleus is essential to sustain efficient interferons (IFNs) signaling and antiviral functions. Therefore, it is important to clarify how pY701-STAT1 levels are positively regulated in the nucleus. Here, we demonstrated for the first time that IFNs-induced pY701-STAT1 largely raised STAT1 ubiquitination, and ubiquitinated-pY701-STAT1 is located predominantly in the nucleus and regulated by proteasome-dependent degradation. In IFNs signaling, the deubiquitinase USP2a translocates into the nucleus and binds to pY701-STAT1. USP2a positively regulates pY701-STAT1 levels through inhibiting its K48-linked ubiquitination and degradation. Importantly, USP2a sustains all three classes of IFNs-mediated signaling and antiviral function. Our studies uncover an important positive mechanism in the nucleus by which IFNs can execute efficient antiviral signaling and functions.

## Introduction

The interferons (IFNs) family of cytokines plays a critical role in the defense against viral infection and regulating innate immunity. Three classes of IFNs (type I, II, and III) have been identified, all of which exert their activity by activating a signaling cascade comprised of Janus kinases (Jak) and signal transducers and activators of transcription (STAT) [1,2]. In this cascade, activated STAT is a key component which regulates IFNs function by inducing the expression of hundreds of IFNs-stimulated genes (ISGs). Amongst the seven members of the STAT family, STAT1 (S1 Table) regulates all three classes of IFNs signaling. In the case of type I-IFN (IFNα/β), IFNα/β induces STAT1 phosphorylation at Tyr701 site (pY701-STAT1). In so doing, STAT1 becomes activated and forms a transcriptional complex [3], which then translocates into the nucleus to induce ISGs expression by binding to the IFNs-stimulated response element (ISRE) located within ISGs promoters [1,2]. Therefore, activated STAT1 signaling largely determines the magnitude and duration of the IFNs signaling and antiviral efficacy.

Previous studies concerning the regulation of STAT1 signaling have been primarily focused on total STAT1. Different posttranslational modifications of total STAT1, such as methylation [4,5], acetylation [6,7,8,9], sumoylation [10,11] and ubiquitination [12,13], have been suggested as means of regulating STAT1 signaling. However, a detailed mechanistic understanding of each is unknown. With regards to ubiquitination modification specifically, the role cytokines play in inducing STAT1 ubiquitination, as well as how STAT1 ubiquitination can regulate subsequent cytokine signaling remain poorly understood. As a matter of fact, total STAT1 protein levels are relatively stable over the course of a couple of hours even under conditions of cytokine stimulation. Thus, STAT1 signaling induced by cytokines (e.g. IFNs) at early stages may not be significantly affected by changes to total STAT1 ubiquitination and protein levels.

As previously mentioned, pY701-STAT1 delivers activated IFNs signaling to the nucleus in order to produce effectors of IFNs function. Therefore, one can understand that STAT1 signaling is actually largely dependent on the level of pY701-STAT1 rather than total STAT1. Given that high levels of pY701-STAT1 are achieved within several minutes of IFNs treatment, it is necessary to uncover how pY701-STAT1 is regulated delicately.

Prevailing thought holds that pY701-STAT1 levels are controlled mainly by dephosphorylation [14,15,16], and the question can be asked as to whether or not pY701-STAT1 protein is directly regulated by other negative mechanisms. Until this point, the question has remained largely unexplored. Recent reports show that STAT1 ubiquitination is regulated by the E3 ligase Smurf1 [17] and the deubiquitinase USP13 [18] (S1 Table). However, both Smurf1 and USP13 do not interact with pY701-STAT1 and ubiquitination regulation of STAT1 by them is independent of IFNs treatment [17,18]. Thus, we sought to determine whether pY701-STAT1 could be directly regulated by the ubiquitin-proteasome system. If so, how ubiquitination modification might regulate pY701-STAT1 to control IFNs signaling and antiviral function.

Here we reveal that IFNs-induced pY701-STAT1 largely raised STAT1 ubiquitination. Moreover, ubiquitinated-pY701-STAT1 accumulated in the nucleus and was tightly regulated by the ubiquitin-proteasome system. Furthermore, we identified the deubiquitinase USP2a as an important player, showing that it translocated into the nucleus and bound to pY701-STAT1. USP2a stabilized pY701-STAT1 by removing K48-linked ubiquitin chains. More importantly, we clarify that USP2a enhanced all three types of IFNs induced signaling and antiviral defenses. Collectively, our findings could provide the mechanistic insight into the development of enhanced IFNs therapeutic efficacy against viral infections.

## Results

### IFN-induced pY701-STAT1 raises STAT1 ubiquitination largely, and ubiquitin-proteasome degradation controls pY701-STAT1 levels

**Fig 1:**
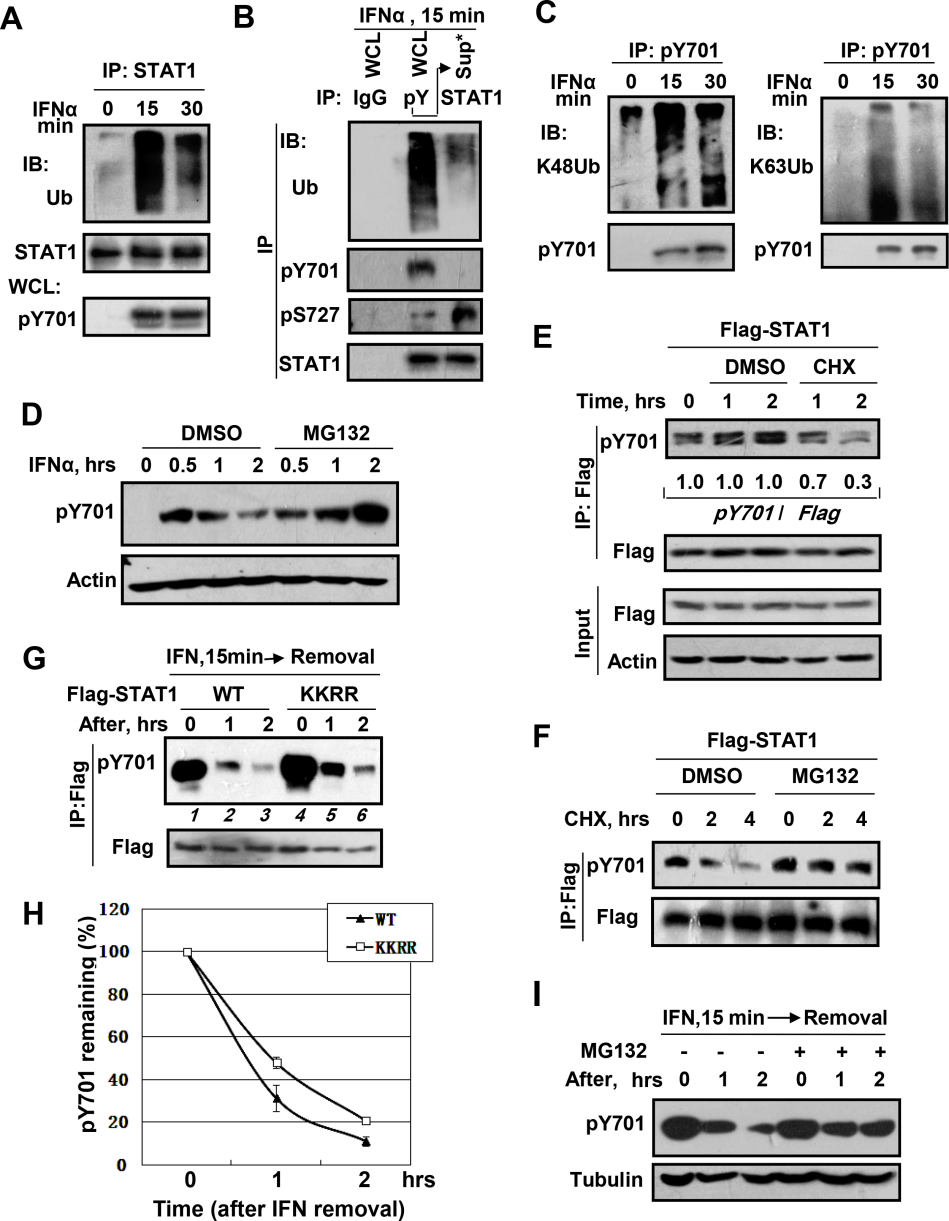
pY701-STAT1 is the major form of ubiquitinated-STAT1 induced by type-I IFN, and ubiquitin-proteasome degradation controls pY701-STAT1 levels largely. (A) 293T cells were treated with IFNα (1,000 IU/ml) as indicated following with MG132 (10 μM) pretreatment for 1 hr. STAT1 proteins were immunoprecipitated and analyzed by indicated antibodies. (B) 293T cells were stimulated by IFNα (1,000 IU/ml) for 15 min. pY701-STAT1 proteins were separated by immunoprecipitation using pY701 antibody. The supernatant from pY701-STAT1-immunoprecipitation was subjected to STAT1 immunoprecipitation using STAT1 antibody. Then levels of ubiquitinated-STAT1, pY701-STAT1 and total STAT1 were analyzed by indicated antibodies. (C) 293T cells were pretreated with MG132 (10 μM) for 1 hr, and then treated with IFNα (1,000 IU/ml) for 15 min and 30 min. pY701-STAT1 proteins were immunoprecipitated and analyzed by indicated antibodies. (D) 293T cells were pretreated with DMSO or MG132 (10 μM) for 2 hrs, and then were treated with IFNα (1,000 IU/ml) as indicated. pY701-STAT1 levels were analyzed by pY701-STAT1 antibody. (E) 293T cells transfected with Flag-STAT1 were treated with DMSO or CHX (50 μM) as indicated. Flag-STAT1 proteins were immunoprecipitated and pY701-STAT1-Flag levels were analyzed as indicated. Whole cell lysates (WCL) was utilized to determine total Flag-STAT1 levels as indicated. (F) 293T cells transfected with Flag-STAT1 were treated with CHX (50 μM) for indicated times together with DMSO or MG132 (10 μM). Flag-STAT1 proteins were immunoprecipitated, and levels of pY701-STAT1-Flag and total Flag-STAT1 were analyzed by indicated antibodies. (G) 293T cells transfected with Flag-STAT1-wild type (WT) or Flag-STAT1-K410,413R (KKRR) were stimulated with IFNα (1,000 IU/ml) for 15 min, then IFNα was removed by washing twice. Cells were further incubated as indicated times. Flag-STAT1 proteins were immunoprecipitated by Flag antibody, and levels of pY701-STAT1-Flag and total Flag-STAT1 were analyzed using indicated antibodies. (H) Quantification of pY701-STAT1-Flag protein levels from (G) (Normalized to total Flag-STAT1). (I) 293T cells preteated with MG132 (10 μM) for 2 hrs were treated with IFNα (1,000 IU/ml) for 15 min, then IFNα was removed. Cells were further incubated as indicated. pY701-STAT1 levels were detected by immunoblotting.

To address the roles of ubiquitination modification in regulating pY701-STAT1, we firstly examined whether type-I IFN could induce STAT1 ubiquitination. Treatment of cells with IFNα induced significant STAT1 ubiquitination (Fig 1A). It has previously been reported that IFNs can induce phosphorylation of STAT1 at Tyr701, Ser727, Ser708 [19], and other sites. Therefore, we questioned whether pY701-STAT1 could undergo ubiquitination modification. Immunoprecipitation with a pY701 antibody was used to separate IFNα-induced pY701-STAT1 from cell lysates. The supernatant resulting from this pY701-immunoprecipitation was then subjected to STAT1 immunoprecipitation using a STAT1 antibody. Interestingly, we found that pY701-STAT1 proteins were significantly ubiquitinated (Fig 1B). Intriguingly, when compared to other STAT1 forms (e.g. pS727-STAT1), pY701-STAT1 could be the predominant form of ubiquitinated STAT1 (Fig 1B). To further demonstrate that pY701-STAT1 is capable of undergoing robust ubiquitination modification, Flag-STAT1-wild type (WT) and–Y701F (YF) plasmids were transfected into cells. The levels of ubiquitination of Flag-STAT1-WT/YF were analyzed by immunoblotting. Results indicated that IFNα was capable of inducing much higher levels of ubiquitination in STAT1-WT when compared to STAT1-YF mutant (S1A Fig). Furthermore, IFNα induced both K48-linked and K63-linked ubiquitination of pY701-STAT1 (Fig 1C). Collectively, these results suggest that IFN-induced pY701-STAT1 was able to significantly raise STAT1 ubiquitination.

We next asked whether ubiquitination modification could promote pY701-STAT1 degradation. We found that in the presence of the proteasome inhibitor MG132, type-I IFN (IFNα)-induced pY701-STAT1 was significantly increased (Fig 1D). It has been known that overexpression of Flag-STAT1 can also produce pY701-Flag-STAT1 independent of IFNs treatment as reported by other groups and shown by our data (S1B Fig). After treatment of cells with the protein translation inhibitor cycloheximide (CHX), levels of pY701-Flag-STAT1 were also rapidly downregulated (Fig 1E), although we did not observe a change in total Flag-STAT1 levels (Fig 1E, bottom panel). STAT1 is a relatively stable protein, which is nearly unchanged even under conditions of 24 hours treatment with CHX (S2A Fig, left lanes). Here, our results suggest that STAT1 protein becomes unstable once it is phosphorylated at Tyr701 and ubiquitinated. In the presence of MG132, pY701-Flag-STAT1 levels were quite stable even if CHX treatment time was extended to 4 hours (Fig 1F). In conjunction with our previous results showing that pY701-STAT1 *per se* undergoes ubiquitination modification, these results suggest that the pY701-STAT1 form is directly regulated by ubiquitination and proteasome-dependent degradation.

Dephosphorylation of STAT1 by phosphatases TC-PTP and SHP-2 (S1 Table) has been thought to be an important negative regulatory mechanism of pY701-STAT1 [6,16]. It has been reported that STAT1 dephosphorylation correlates with its acetylation at both K410 and K413 sites [6,8]. IFN-induced pY701-STAT1 translocates into the nucleus, where it binds to the acetyl-transferase CBP [6,20,21]. CBP-mediated acetylated STAT1 recruits phosphatases, resulting in dephosphorylation [6]. To assess whether ubiquitin-proteasome degradation is important for pY701-STAT1 regulation, we constructed STAT1-K410, 413R (KKRR) mutant, which does not have dysfunction in nuclear import (S1C Fig). It has been proved that STAT1-KKRR mutant cannot be acetylated and cannot bind to TC-PTP. Therefore it cannot be dephosphorylated by TC-PTP [6]. Cells transfected with Flag-STAT1 wild type (WT) or KKRR were treated with IFNα for 15 min after which IFNα was removed. As expected, pY701-STAT1-KKRR levels were higher than pY701-STAT1-WT (Fig 1G, lane 1 vs lane 4). This result is consistent with a previous report that demonstrated the inability of KKRR to be dephosphorylated. As shown in Fig 1G, pY701-STAT1-WT levels decreased rapidly after IFNα removal, which may be due to both dephosphorylation and ubiquitin-proteasome degradation. Interestingly, levels of pY701-STAT1-KKRR were also downregulated considerably, although not as rapidly as those in pY701-STAT1-WT (Fig 1G and 1H). Therefore, we speculated that pY701-STAT1 could be downregulated through not only dephosphorylation mechanisms. However, the effect of K410, K413 sites of STAT1 on TC-PTP-mediated dephosphorylation remains controversial [22]. Moreover, the STAT1-KKRR mutant cannot inhibit dephosphorylation on pY701-STAT1 performed by other phosphatases. Therefore, we further used a pan phosphatases inhibitor NSC87877, which is capable of inhibiting several phosphatases including SHP-1/2, PTP1B, He-PTP and CD45. Similarly, we found that pY701-STAT1 was still downregulated to a large extent when cells were treated with this inhibitor (S2B Fig). More importantly, when the proteasome inhibitor MG132 was used to inhibit the effect of ubiquitin-proteasome system, we found that downregulation of pY701-STAT1 was largely blocked (Fig 1I and S1D Fig), although pY701-STAT1 levels were still reduced to some extent which could be due to a dephosphorylation effect (Fig 1I, lane 4–6). Thus, our results suggest that the ubiquitin-proteasome system plays an important role in regulating pY701-STAT1.

### Ubiquitinated-pY701-STAT1 accumulates in the nucleus

**Fig 2:**
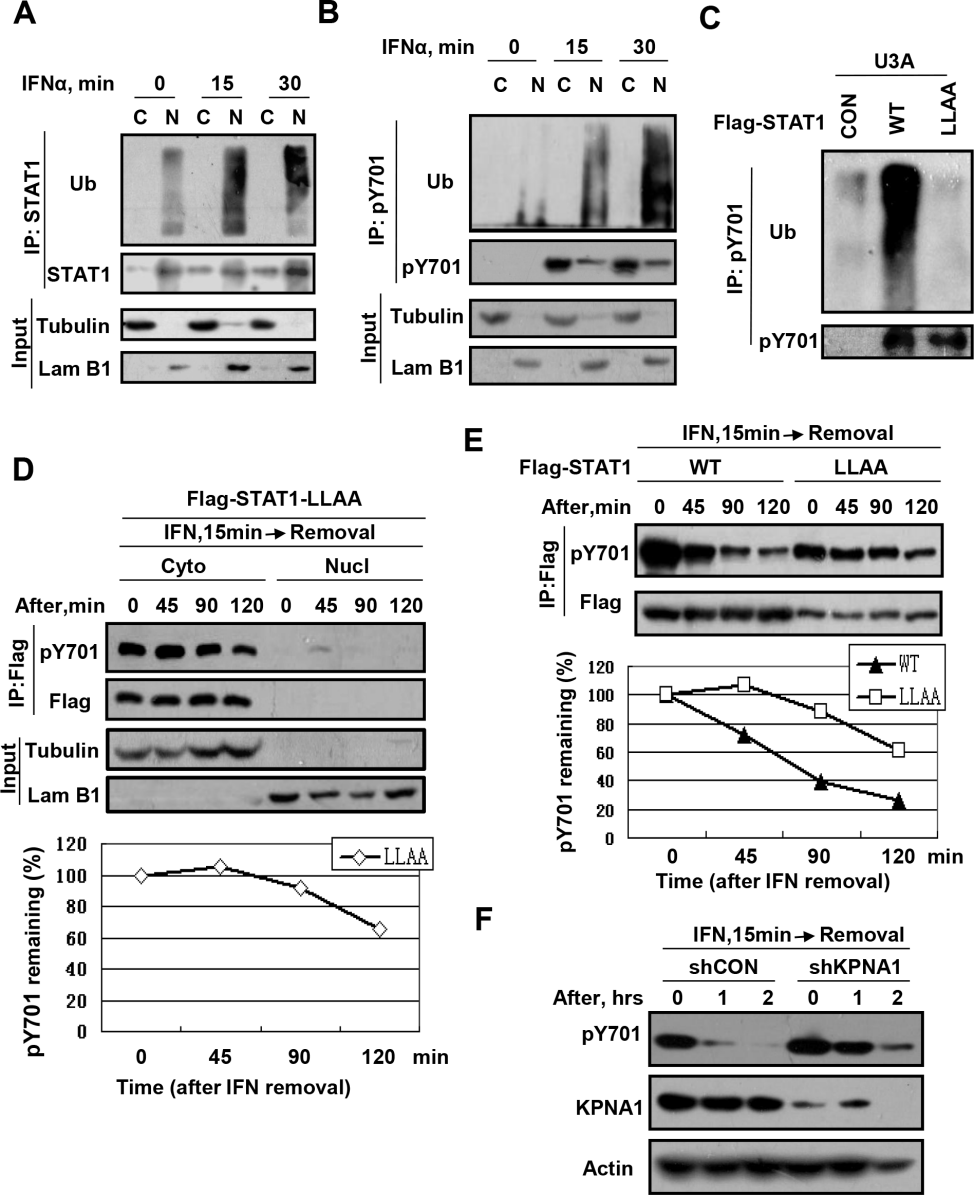
Ubiquitinated-pY701-STAT1 accumulates in the nucleus. (A) 293T cells were treated with IFNα (1,000 IU/ml) for 15 min and 30 min. STAT1 ubiquitination was analyzed using the proteins from the cytoplasm and the nucleus as indicated. (B) 293T cells were treated as in (A). The proteins from the cytoplasm and the nucleus were analyzed by indicated antibodies. (C) U3A cells were transfected with Flag-STAT1-WT or Flag-STAT1-LLAA. pY701-Flag-STAT1 was immunoprecipitated using specific antibody, and immunoblotting was performed as indicated. (D) 293T cells transfected with Flag-STAT1-LLAA were stimulated with IFNα (1,000 IU/ml) for 15 min, then IFNα was removed. The proteins from the cytoplasm and the nucleus were analyzed as indicated antibodies. pY701-STAT1-Flag levels were quantificated. (E) 293T cells transfected with Flag-STAT1-WT or -LLAA were stimulated with IFNα (1,000 IU/ml) for 15 min, then IFNα was removed. The level of pY701-STAT1-Flag-WT/LLAA from whole cell lysates were analyzed and quantificated as indicated. (F) 293T cells transfected control shRNA (shCON) or KPNA1 shRNA (shKPNA1) were stimulated with IFNα (1,000 IU/ml) for 15 min, then IFNα was removed. The whole cell lysates were analyzed using indicated antibodies.

Given that IFNs-induced pY701-STAT1 translocates into the nucleus to activate ISGs transcription, we next questioned where ubiquitinated-pY701-STAT1 proteins are predominantly located (e.g. cytoplasm or nucleus). To this end, cells were treated with IFNα for either 15 min or 30 min. The proteins from the cytoplasm and the nucleus were subjected to the analysis of STAT1 ubiquitination. Interestingly, the data showed that IFN-stimulated ubiquitinated-STAT1 accumulated mainly in the nucleus (Fig 2A), which is consistent with another observation of ours (S2C Fig). Furthermore, pY701-STAT1 proteins were immunoprecipitated and immunoblotted with an ubiquitin antibody. Consistent with the locations of IFNs-induced ubiquitinated-STAT1, we found that most of ubiquitinated-pY701-STAT1 proteins also accumulated in the nucleus (Fig 2B). To provide further evidence for the nuclear location of ubiquitinted-pY701-STAT1, we blocked the nuclear import of STAT1 and then analyzed the ubiquitination levels of pY701-STAT1. Previous work has shown that the Flag-STAT1-L407,409A (LLAA) mutant, which can be tyrosine phosphorylated to a similar degree as Flag-STAT1-wild type (WT), was unable to enter the nucleus even if under conditions of IFNs stimulation [23]. U3A cells (STAT1-deficient) were transfected with Flag-STAT1-WT or Flag-STAT1-LLAA. pY701-Flag-STAT1 was immunoprecipitated and the ubiquitination level of pY701-Flag-STAT1 was analyzed. Our results showed that Flag-STAT1-LLAA underwent much less ubiquitination when compared to Flag-STAT1-WT, despite the fact that they had the same pY701-STAT1 levels (Fig 2C). This result suggests that blocking nuclear import of pY701-STAT1 inhibits pY701-STAT1 ubiquitination.

Taken together, the aforementioned results suggest that ubiquitinated-pY701-STAT1 could be regulated by a degradative mechanism driven by the ubiquitin-proteasome system in the nucleus. To try to provide some evidence for this hypothesis, cells transfected with Flag-STAT1-LLAA were stimulated with IFNα for 15 min, after which the IFNα was removed. As expected, Flag-STAT1-LLAA was located predominantly in the cytoplasm even under conditions of IFNs stimulation (Fig 2D). To our surprise, pY701-STAT1-LLAA was relatively stable within 90 min after IFNs removal, and was only slightly downregulated even at 120 min after IFNs removal (Fig 2D). Next, the whole cell lysates were analyzed. Likewise, Flag-STAT1-LLAA was markedly more stable after IFNs removal, when compared to Flag-STAT1-WT (Fig 2E). Furthermore, we used a knockdown strategy to block the nuclear import of pY701-STAT1. KPNA1 (importin-α5) (S1 Table) has been shown to be responsible for the nuclear import of pY701-STAT1 [24,25]. We found that knockdown of KPNA1 remarkably inhibited the downregulation of pY701-STAT1 (Fig 2F). To directly observe ubiquitin-proteasome degradation of nuclear pY701-STAT1, we used a pY701-STAT1 specific antibody to analyze IFN-induced STAT1 nuclear import and the colocalization of pY701-STAT1 with the proteasome in the nucleus using the immunofluorescence staining. Our results showed that IFNα-induced pY701-STAT1 clearly colocalized with the proteasomal marker PA28β in the nucleus (S3 Fig). The above data suggest that pY701-STAT1 could be ubiquitinated and regulated in the nucleus via the ubiquitin-proteasome system, although it is possible that the cytoplasmic proteasome also contributes to pY701-STAT1 regulation to some extent.

### Identification of USP2a as the deubiquitinase for pY701-STAT1 regulation

**Fig 3:**
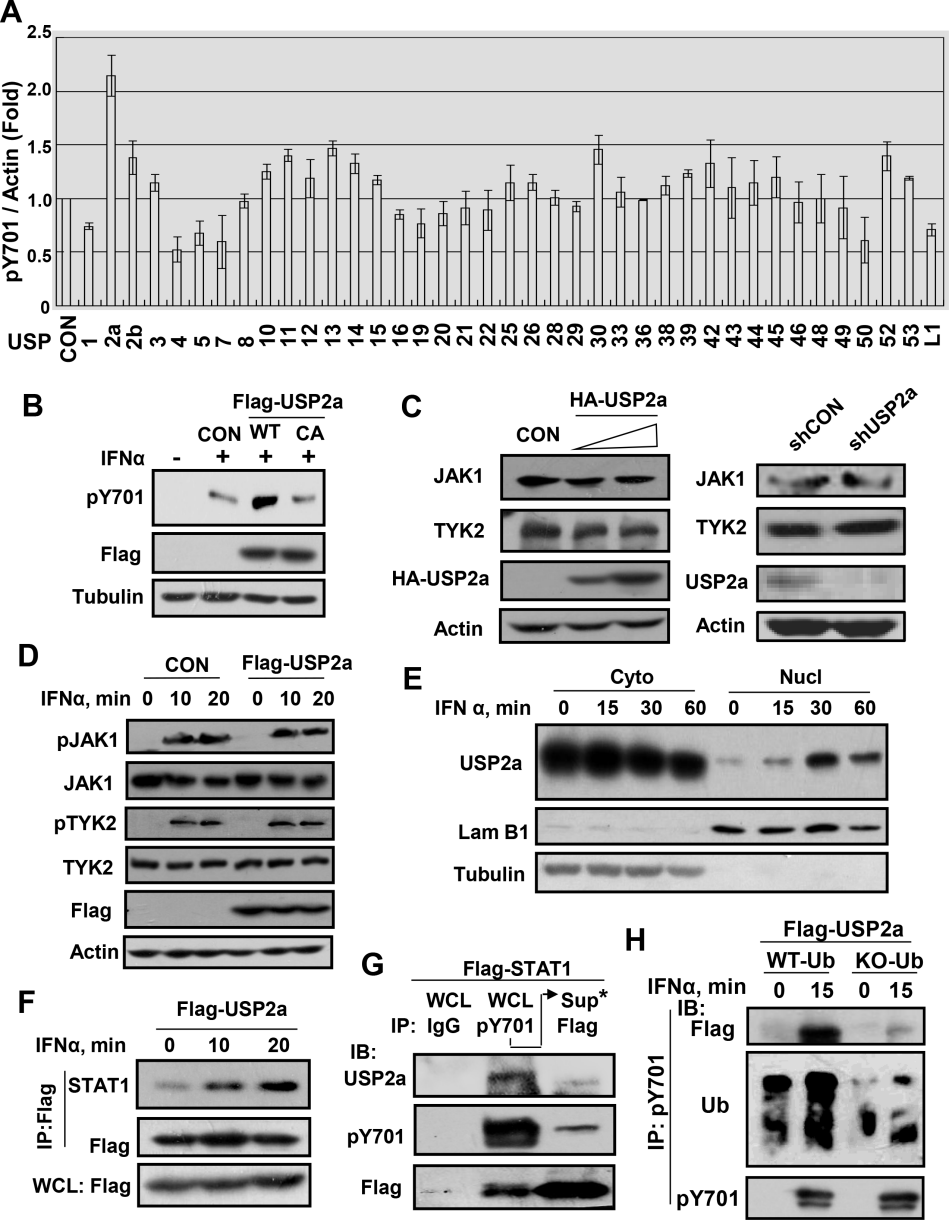
USP2a translocates into the nucleus, and binds to pY701-STAT1 to upregulate pY701-STAT1 levels in type-I IFN signaling. (A) The identification of USP2a as the ubiquitin-specific protease (USP) which increases the level of IFNα-induced pY701-STAT1 by overexpression of USPs library in 293T cells followed by IFNα (1,000 IU/ml) treatment for 30 min. (B) 293T cells transfected with Flag-USP2a-WT or–C276A were stimulated with IFNα for 15 min. pY701-STAT1 was analyzed as indicated. (C) 293T cells were transfected with HA-USP2a or shUSP2a. The immunoblotting was performed as indicated. (D) 293T cells transfected with or without Flag-USP2a were treated with IFNα (1,000 IU/ml) for 10 min and 20 min. The immunoblotting was performed as indicated. (E) 293T cells were treated with IFNα (1,000 IU/ml) for 0, 15, 30 and 60 min. The proteins from the cytoplasm and the nucleus were analyzed by indicated antibodies. (F) 293T cells transfected with Flag-USP2a were treated with IFNα (1,000 IU/ml) for indicated times. Flag-USP2a was immunoprecipitated, and STAT1 levels were analyzed as indicated. (G) 293T cells were transfected with Flag-STAT1. pY701-STAT1 proteins were immunoprecipitated by pY701 antibody. The supernatant from pY701-immunoprecipitation was subjected to Flag-STAT1 immunoprecipitation using Flag antibody. The immunoblotting was performed as indicated. (H) 293T cells transfected with Flag-USP2a, together with WT-Ub or KO-Ub, were treated with IFNα for 15 min. pY701-STAT1 proteins were immunoprecipitated, and immunoblotting was performed as indicated.

To further demonstrate that the ubiquitin-proteasome system does regulate pY701-STAT1 levels, we sought to identify what key component of the ubiquitin-proteasome system may regulate pY701-STAT1 ubiquitination. Although several studies have observed the regulation of STAT1 ubiquitination, all of them were focused on total STAT1. As shown in our data, knockdown of Smurf1, which has been reported as the E3 ligase of total STAT1, had no obvious effect on IFN-induced ubiquitination of pY701-STAT1 (S2D Fig). So far, there are no deubiquitinases that have been identified important for pY701-STAT1 regulation. Therefore, we screened a library of 39 human ubiquitin-specific proteases (USPs) to identify potential candidates [26]. In this screening, overexpression of USP2a, USP13, USP30 and USP52 remarkably increased IFNα-induced pY701-STAT1 levels, whereas other USPs had no effect or fewer effects (Fig 3A). USP13 has been excluded by previous studies. We further found that USP2a (S1 Table), but neither USP30 nor USP52, bound to pY701-STAT1 (S4A Fig). The USP2 family includes two major isoforms, USP2a (69 KDa) and USP2b (45 KDa) [27,28,29]. To further investigate the role of USP2, we turned to a USP2 specific antibody, which can recognize human but not mouse USP2a and USP2b. This specificity was both demonstrated by the manufacturer’s data sheet as well as shown in our data (S5A Fig), we found that USP2a proteins were expressed in many different types of cells, while USP2b proteins were hardly to be detected by immunoblotting in these cell lines (S5B Fig). USP2b protein was undetectable even after 60 minutes of IFNα stimulation (S5C Fig). Therefore, we focused on USP2a to elucidate its possible effects of the ubiquitin-proteasome system on pY701-STAT1.

Given that USP2a upregulates IFNα-induced pY701-STAT1, we sought to determine if the deubiquitinase activity of USP2a is required for pY701-STAT1 regulation. Cells transfected with Flag-USP2a-WT and catalytically inactive Flag-USP2a–C276A (CA) were treated with IFNα. We found that catalytically inactive USP2a-CA was unable to upregulate pY701-STAT1 levels induced by IFNα (Fig 3B), suggesting that USP2a upregulates pY701-STAT1 which is dependent on its deubiquitinase activity. Furthermore, we found that cellular levels of both JAK1 and Tyk2 (S1 Table) were unchanged when USP2a was either overexpressed or knocked down in cells (Fig 3C), suggesting that the deubiquitinase USP2a regulates neither of them. Next, we examined whether USP2a promotes IFN-induced upstream signaling of STAT1, activated p-Tyk2 and p-JAK1. We observed no upregulation in levels of either p-Tyk2 or p-JAK1 (Fig 3D), suggesting that the target of USP2a could be in the downstream of JAK1/Tyk2. In addition, our data showed that USP2a was unable to upregulate the expression of TC-PTP (S4B Fig), which is an important phosphatase of pY701-STAT1. Furthermore, our data showed that knockdown of USP2a was unable to decrease either total STAT1 levels or its stability (S2A Fig). Collectively, the above results suggest that USP2a could be a potential deubiquitinase for pY701-STAT1 regulation.

### IFN induces the nuclear import of USP2a and promotes the binding of USP2a to pY701-STAT1

Given that our above data demonstrated that ubiquitinated-pY701-STAT1 is located largely in the nucleus, we wanted to know whether USP2a protein also exists in the nucleus. Our results showed that USP2a was located in both the cytoplasm and the nucleus even in the absence of IFNs treatment (Fig 3E). More importantly, we found that IFNs treatment promoted the nuclear import of USP2a (Fig 3E). Interestingly, we observed that USP2a nuclear import was inhibited by knockdown of Importin α (S4C Fig), which is an important mediator for protein nuclear import. This suggests that IFN-induced USP2a nuclear import could also be mediated by a member of the importin α family. In addition, the pY701 form of Flag-STAT1-KKRR mutant, which cannot be dephosphorylated by TC-PTP in the nucleus, was also regulated by USP2a knockdown (S4D Fig). Taken together, these results suggest that USP2a can translocate into the nucleus to regulate ubiquitinated-pY701-STAT1 when IFNs induce the nuclear accumulation of ubiquitinated-pY701-STAT1.

Evidence from a co-immunoprecipitation assay showed that IFNα promoted the binding between USP2a and STAT1 (Fig 3F). We next investigated whether it is the pY701-STAT1 form which binds to USP2a. We found that USP2a was able to bind with Flag-STAT1-WT, but not with Flag-STAT1-Y701F (S4E Fig). Furthermore, we then transfected cells with Flag-STAT1, after which pY701-STAT1 and un-pY701-STAT1 were separated using an immunoprecipitation procedure as described above. Our results showed that USP2a interacted with pY701-STAT1, but not other STAT1 forms (Fig 3G). Similarly, USP2a interacted with IFN-induced pY701-STAT1 (S4F Fig), but not pS727-STAT1 (S4G Fig), confirming an interaction between USP2a and pY701-STAT1.

Given that pY701-STAT1 is the major form of ubiquitinated-STAT1, we believed that the binding between the deubiquitinase USP2a and pY701-STAT1 would depend on the ubiquitination of pY701-STAT1. Therefore, KO-Ub (all seven lysines in ubiquitin are mutated to arginines) was overexpressed to competitively inhibit the ubiquitination of pY701-STAT1. We found that overexpression of KO-Ub inhibited the ubiquitinaion of pY701-STAT1 and markedly reduced the binding between USP2a and pY701 (Fig 3H). In summary, our results show that during IFNs signaling, USP2a translocates into the nucleus to interact with ubiquitinated-pY701-STAT1.

### USP2a regulates pY701-STAT1 ubiquitination

**Fig 4:**
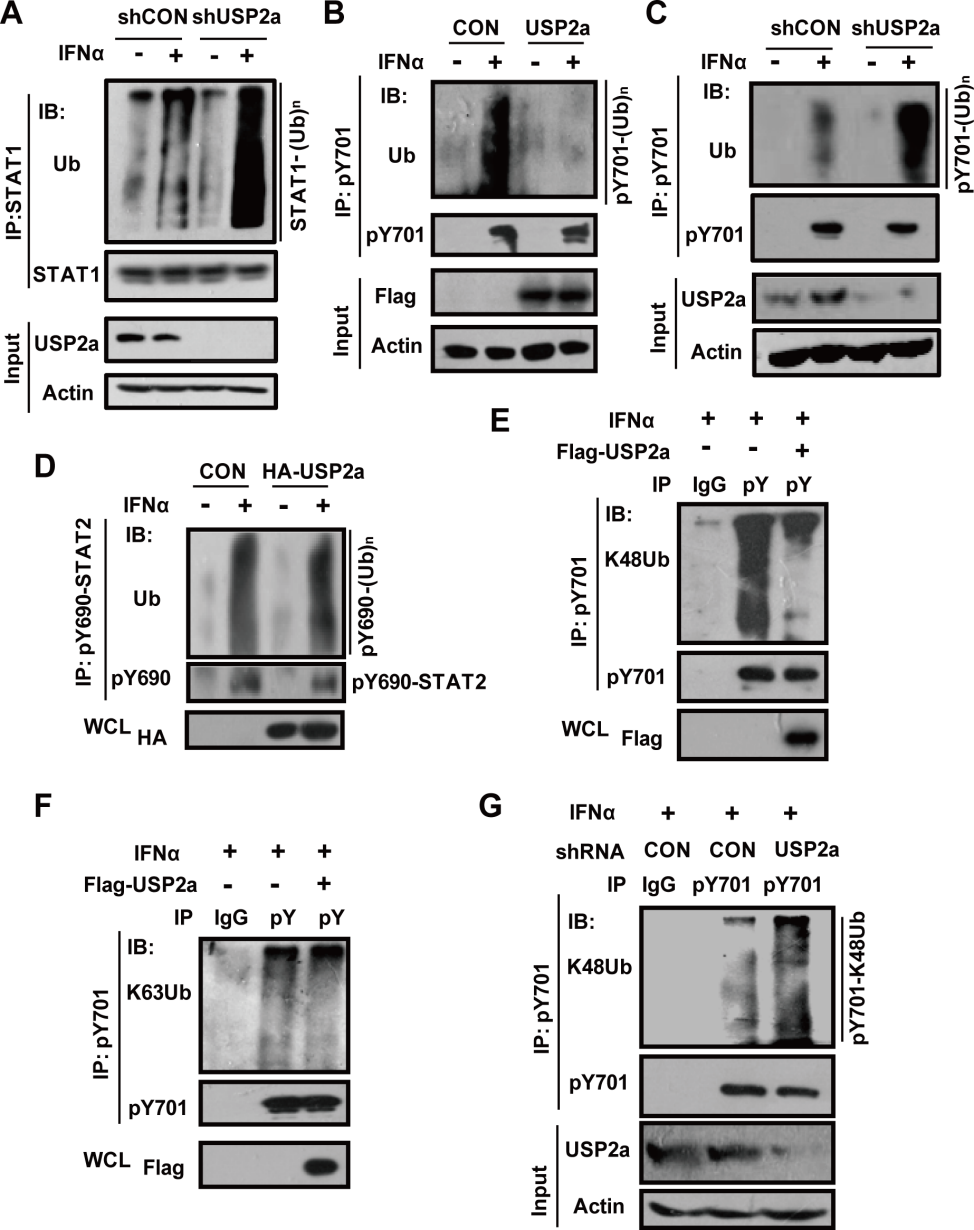
USP2a inhibits K48-linked ubiquitination of pY701-STAT1 in type-I IFN signaling. (A) 293T cells transfected with shCON or shUSP2a were pretreated with MG132 (10 μM) pretreatment for 1 hr, and then treated with IFNα (1,000 IU/ml) as indicated. STAT1 proteins were immunoprecipitated, and ubiquitinated-STAT1 levels were analyzed as indicated. (B) 293T cells transfected with empty vector or Flag-USP2a were pretreated with MG132 (10 μM) pretreatment for 1 hr, and then treated with IFNα (1,000 IU/ml) for 15 min. pY701-STAT1 proteins were immunoprecipitated as indicated, and ubiquitination of pY701-STAT1 was analyzed as indicated. (C) 293T cells transfected with shCON or shUSP2a were treated with IFNα as (B). The immunoblotting was performed as (B). (D) 293T cells transfected with or without HA-USP2a were treated with IFNα for 15 min. pY690-STAT2 proteins were immunoprecipitated by a pY690 antibody, and immunoblotting was performed as indicated. (E, F) 293T cells transfected with or without Flag-USP2a were treated with IFNα (1,000 IU/ml) for 15 min. pY701-STAT1 proteins were immunoprecipitated, and the levels of K48-linked (E) and K63-linked (F) ubiquitination of pY701-STAT1 were analyzed as indicated. (G) 293T cells transfected with shCON or shUSP2a were treated with IFNα for 15 min. pY701-STAT1 proteins were immunoprecipitated by pY701 antibody, and immunoblotting was performed as indicated.

Given that IFNs rapidly induce STAT1 Tyr701 phosphorylation and ubiquitination (Fig 1A), we first examined whether USP2a plays a role in regulating IFNs-induced STAT1 ubiquitination. USP2a knockdown markedly enhanced IFNα-induced STAT1 ubiquitination (Fig 4A). Conversely, USP2a overexpression inhibited IFNα-induced STAT1 ubiquitination (S6A Fig). To further demonstrate the effect of USP2a on pY701-STAT1 ubiquitination, pY701-STAT1 was immunoprecipitated from IFNα-treated cells, and ubiquitination of pY701-STAT1 was analyzed. Results showed that USP2a overexpression significantly decreased IFNα-induced pY701-STAT1 ubiquitination (Fig 4B and S6B Fig). USP2a knockdown increased IFNα-induced pY701-STAT1 ubiquitination (Fig 4C). Furthermore, we want to know whether USP2a also affects STAT2 ubiquitination in IFNs signaling, given that IFNs signaling activates STAT2 (pY690-STAT2), which then forms a heterodimer with STAT1. Interestingly, we found that IFNα remarkably induced ubiquitination of Flag-STAT2 (S6C and S6D Fig). Our data showed that USP2a overexpression did not obviously affect IFNα-induced ubiquitination of Flag-STAT2 (S6C Fig). And knockdown of USP2a did not increase ubiquitination levels of Flag-STAT2 induced by IFNα (S6D Fig). Moreover, we did not observe the obvious effect of USP2a on IFNα-induced ubiquitination of pY690-STAT2 (Fig 4D). Taken together, we think that in IFNs signaling, USP2a contributes to deubiquitination of STAT1 rather than STAT2.

Next, we sought to determine whether USP2a is capable of removing both K48-linked and K63-linked ubiquitin chains from pY701-STAT1. We found that USP2a overexpression significantly reduced IFNα-induced K48-linked ubiquitination of pY701-STAT1 (Fig 4E). Moreover, knockdown of USP2a increased IFNα-induced K48-linked ubiquitination of pY701-STAT1 (Fig 4G). However, USP2a did not noticeably affect IFNα-induced K63-linked ubiquitination of pY701-STAT1 (Fig 4F). Taken together, during IFNs signaling USP2a significantly inhibits K48-linked, but not K63-linked, ubiquitination of pY701-STAT1.

### USP2a sustains cellular levels of pY701-STAT1

**Fig 5:**
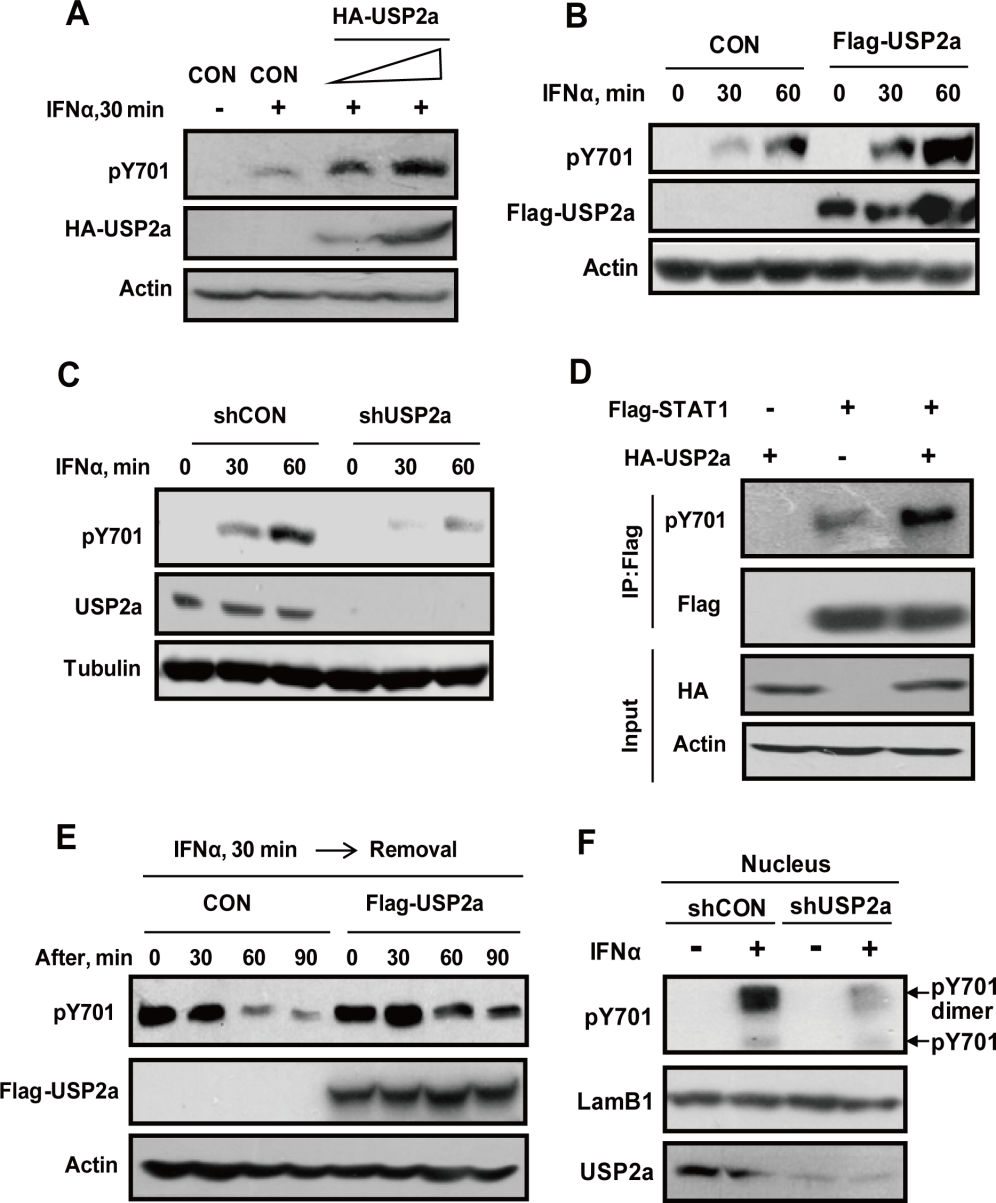
USP2a sustains pY701-STAT1 levels in cells. (A) 293T cells transfected with or without HA-USP2a were treated with IFNα (500 IU/ml) for 30 min. The levels of pY701-STAT1, HA-USP2a and β-actin were immunoblotted as indicated. (B) HT1080 cells transfected with or without Flag-USP2a were treated with IFNα (500 IU/ml) for 30 min and 60 min, and immunoblotting was performed as indicated. (C) 293T cells transfected with shCON or shUSP2a were treated with IFNα (500 IU/ml) for 30 min and 60 min, and immunoblotting was performed as (B). (D) 293T cells were transfected with Flag-STAT1 and (or) HA-USP2a as indicated. Flag-STAT1 proteins were immunoprecipitated by Flag antibody, and pY701-STAT1-Flag levels were analyzed using indicated antibody. (E) 293T cells transfected with empty vector or Flag-USP2a were stimulated with IFNα (1,000 IU/ml) for 30 min. IFNα was removed and then cells were incubated for indicated times. The levels of pY701-STAT1, Flag-USP2a and β-actin were immunoblotted as indicated. (F) 293T cells transfected with shCON or shUSP2a were treated with IFNα for 30 min. The nuclear proteins were separated, and then were subjected to native-PAGE analysis by indicated antibodies.

We next asked whether USP2a could regulate pY701-STAT1 levels. As shown in Fig 5A, USP2a overexpression markedly increased pY701-STAT1 levels following IFNα stimulation (Fig 5A). Consistently, when cells were treated with IFNα for a continuous 30 or 60 min, pY701-STAT1 levels were increased by overexpression of Flag-USP2a (Fig 5B and S6E Fig). USP2a knockdown resulted in decreased levels of pY701-STAT1 in IFNα signaling (Fig 5C). Similarly, Flag-STAT1 was overexpressed to produce pY701-Flag-STAT1. Results clearly showed that in the absence of IFNα treatment, USP2a overexpression significantly increased pY701-Flag-STAT1 levels (Fig 5D), confirming the effect of USP2a on pY701-STAT1. Given that the binding of IFNα to its receptor induces pY701-STAT1, it is possible that intracellular accumulation of pY701-STAT1 induces a negative feedback signal to regulate the binding between IFNα and its receptors, and therefore affects cellular levels of pY701-STAT1. To rule out this possibility, IFNα was removed after a short stimulation, and then the reduction course of pY701-STAT1 was measured. Apparently, USP2a overexpression inhibited the downregulation of pY701-STAT1 (Fig 5E). Hence, these findings strongly indicate that USP2a can regulate cellular levels of pY701-STAT1.

Given that IFNα-induced dimers of pY701-STAT1 translocate into the nucleus, we next determined the effect of USP2a on either pY701-STAT1 monomers or dimers. Using native PAGE gel, we found that nuclear pY701-STAT1 existed mostly in its dimer form. Knockdown of USP2a significantly decreased nuclear pY701-STAT1 dimers induced by IFNα (Fig 5F). USP2a overexpression increased nuclear pY701-STAT1 dimers (S6F Fig). Collectively, these results suggest that USP2a sustains nuclear levels of pY701-STAT1 dimers.

Taken together, we think that in IFNs signaling, pY701-STAT1 translocates into the nucleus, and nuclear pY701-STAT1 undergoes ubiquitination modification to control excessive nuclear pY701-STAT1-activated signaling. As a balance, ubiquitinated-pY701-STAT1 is capable of recruiting USP2a to deubiquitinate pY701-STAT1, which maintains appropriate nuclear pY701-STAT1 levels. As to those pY701-STAT1 retaining in the cytoplasm, we speculate that they are not ubiquitinated in order to provide enough resources for nuclear pY701-STAT1 signaling.

### USP2a promotes antiviral defenses mediated by type-I IFN signaling

**Fig 6:**
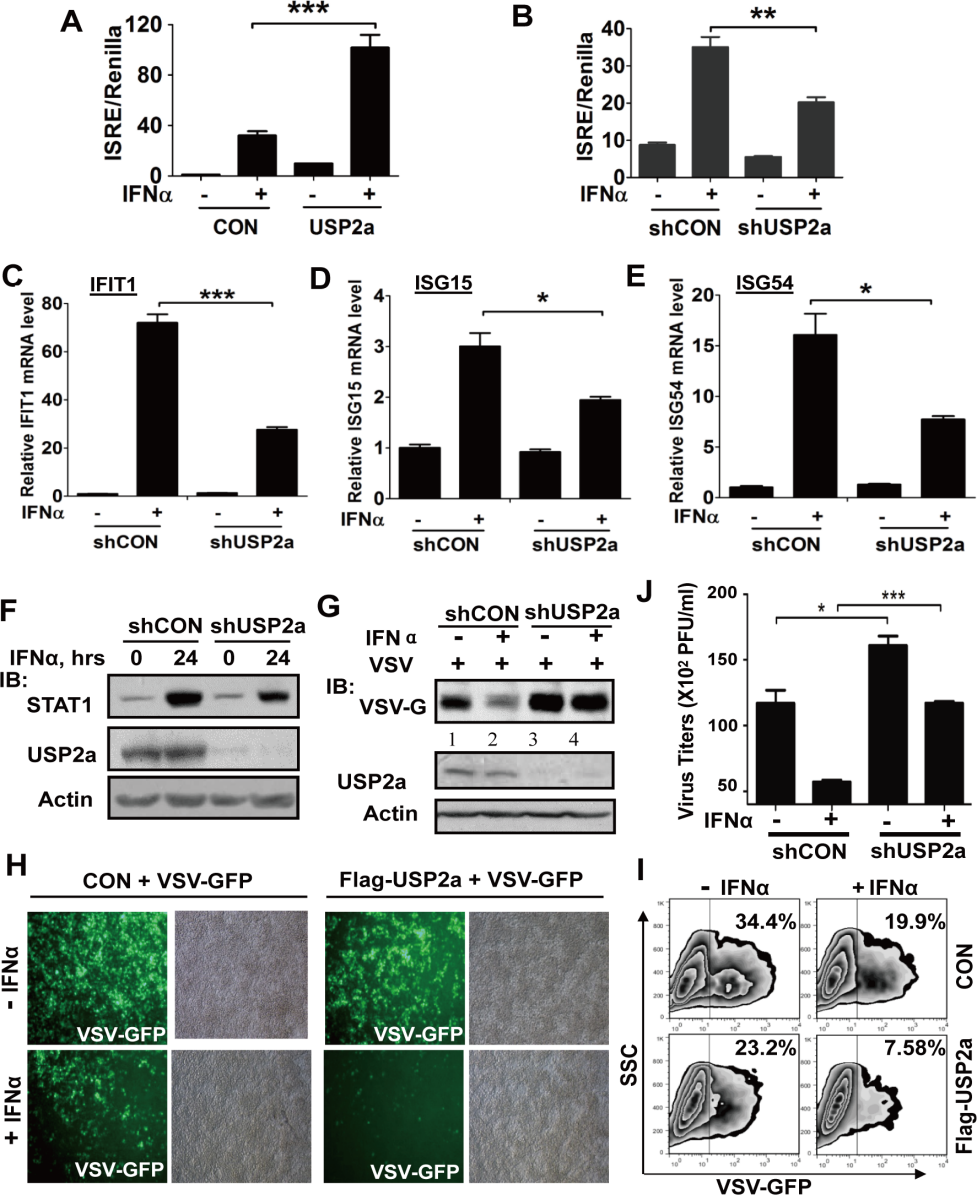
USP2a promotes antiviral defenses mediated by type-I IFN signaling. (A) 293T cells were transfected with empty vector or Flag-USP2a, together with ISRE-Luc and Renilla. The luciferase activity was measured after IFNα (1,000 IU/ml) treatment for 12 hrs. (B) 293T cells were transfected with shCON or shUSP2a, together with ISRE-Luc and Renilla. The luciferase activity was measured 12 hr after IFNα (1,000 IU/ml) treatment. (C, D, E) 293T cells were transfected with control shRNA or USP2a shRNA. Cells were collected after IFNα (1,000 IU/ml) treatment for 9 hrs and the mRNA levels of IFIT1 (C), ISG15 (D) and ISG54 (E) were analyzed by quantitative RT-PCR. (F) 293T cells transfected with shCON or shUSP2a were treated with IFNα (1,000 IU/ml) for 24 hrs, and the immunoblotting was performed as indicated. (G) 293T cells transfected with shCON or shUSP2a were treated with IFNα (50 IU/ml) overnight, and then cells were challenged by VSV (MOI = 1.0). After 20 hrs, the levels of VSV-G, USP2a andβ-actin were immunoblotted as indicated. (H) 293T cells transfected with empty vector or Flag-USP2a were treated with IFNα (50 IU/ml) overnight, and then cells were challenged by VSV-GFP (MOI = 0.5). After 24 hrs, VSV-GFP levels were detected by fluorescence. (I) VSV-GFP levels in experiment (H) were analyzed by FACS. (J) 293T cells transfected with shCON or shUSP2a were treated with IFNα (50 IU/ml) overnight, and then cells were challenged by VSV-GFP (MOI = 0.5). After 24 hrs, cell culture supernatant was collected, and a plaque assay was used for analysis of infectious viral titers. *p<0.05, **p<0.01, ***p<0.001.

Since pY701-STAT1 levels are critical to type-I IFN signaling pathway, we speculated that USP2a could regulate the type-I IFN signaling and its antiviral function. The cis-reporting construct, ISRE-Luciferase, has been widely used to quantify the magnitude of IFNs signaling. We found that USP2a overexpression significantly increased IFNα-induced ISRE-Luciferase activity (Fig 6A), whereas USP2a knockdown remarkably decreased ISRE-Luciferase activity induced by IFNα (Fig 6B). These results demonstrated that USP2a positively regulates the IFNs-induced JAK-STAT signaling pathway.

To directly analyze the effect of USP2a on IFNs-induced gene activation, the mRNA levels of three ISGs including IFIT1, ISG15, and ISG54 (S1 Table) were determined. USP2a knockdown markedly decreased mRNA levels of all three ISGs induced by IFNα (Fig 6C, 6D and 6E). Furthermore, we investigated ISG expression at the protein level. STAT1 protein is usually used to assess IFNs-activated signaling as a representative ISG. We found that USP2a knockdown substantially attenuated IFNs-induced increase of STAT1 proteins (Fig 6F). Consistently, USP2a overexpression enhanced IFNα-stimulated STAT1 expression (S7A Fig). Altogether, these results show that USP2a promotes IFNα-induced expression of ISGs.

Next, we explored the effect of USP2a on type-I IFN antiviral function. Vesicular stomatitis virus (VSV) has been used as a very sensitive viral model to assess IFNs antiviral function. Pretreatment of cells with IFNα enhanced cellular antiviral defenses and therefore decreased the levels of VSV-G, a VSV-encoded protein. USP2a knockdown markedly inhibited IFNα-induced antiviral defenses (Fig 6G). This result is consistent with the observation that cellular antiviral ability under basal conditions of IFNs secretion was significantly enhanced by USP2a-WT overexpression, but not by the catalytically inactive USP2a-C276A mutant (S7B Fig). This result cannot be due to the increase of IFNs production by USP2a overexpression in cells infected with viruses, since Flag-USP2a overexpression actually decreased IFNβ production induced by Sendai Virus (SeV) to some extent (S7C Fig). Consistently, knockdown of USP2a inhibited cellular antiviral defense under basal IFNs-secreted environment (Fig 6G, lane 1 vs lane 3). Furthermore, U3A cells (STAT1-deficient) were used to demonstrate that the effect of USP2a on cellular antiviral activity occurs through IFNs-STAT1 signaling. 2fTGH cells are the parental cells of U3A, which have wild type STAT1 and relatively high levels of autocrine IFNs. Our results showed that the lack of IFNs-STAT1 signaling abolished the effect of USP2a on cellular antiviral ability (S8A Fig).

To better observe the effect of USP2a on IFN-induced antiviral function, a VSV-GFP construct was used to infect cells. As expected, IFNα treatment decreased VSV-GFP infection as shown by decreased GFP signal. Overexpression of USP2a remarkably enhanced IFNα-induced antiviral defenses (Fig 6H). Cells were then collected and subjected to FACS analysis. The data confirmed that USP2a overexpression downregulated the percentage of VSV-GFP-positive cells under IFNα treatment (Fig 6I). Thus, our data demonstrate that USP2a promotes type-I IFN-induced antiviral function.

### USP2a enhances type-II and type-III IFNs-mediated signaling and antiviral function

**Fig 7:**
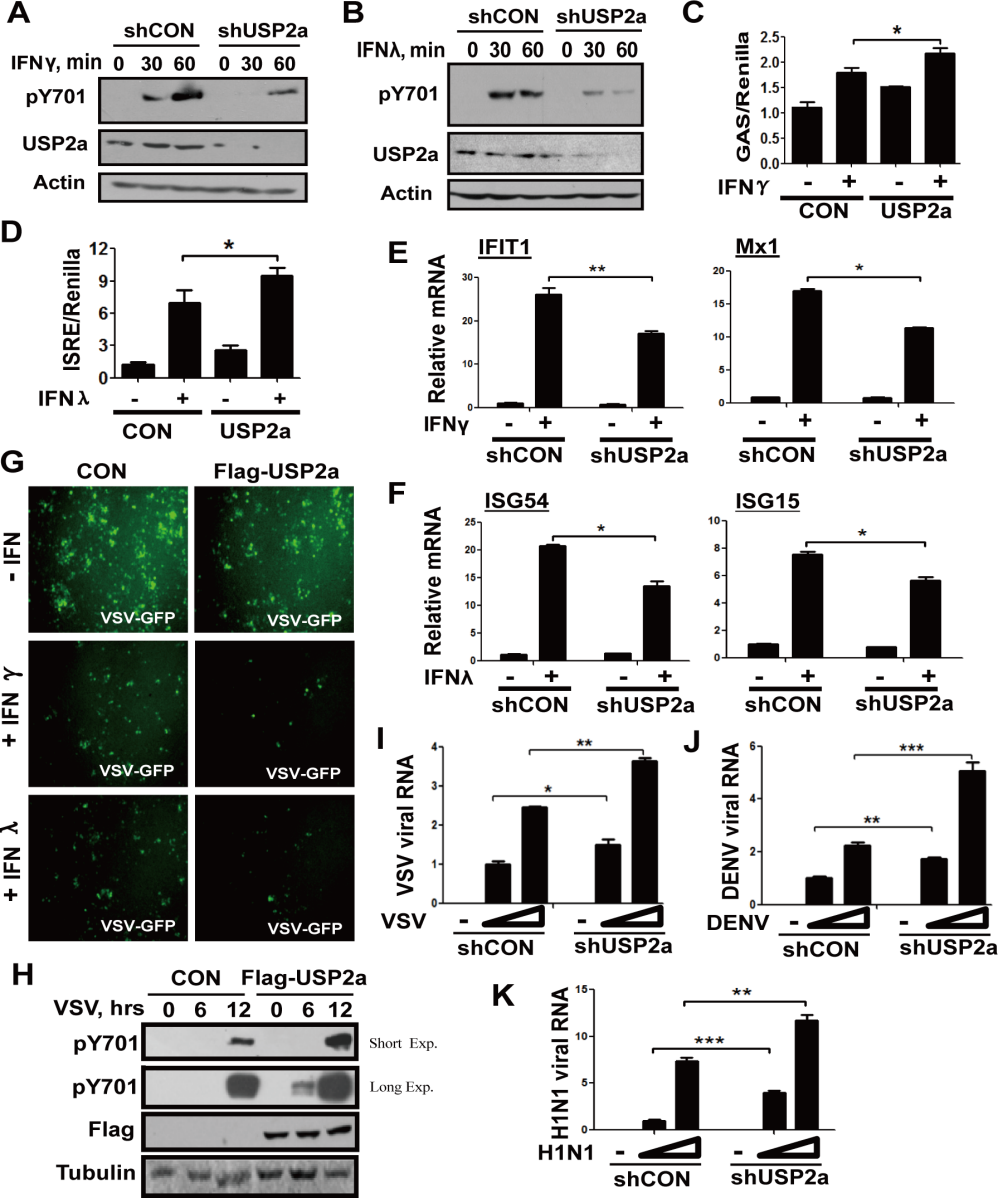
USP2a enhances type-II and type-III IFNs-mediated signaling and antiviral efficacy. (A, B) 293T cells transfected with empty vector or Flag-USP2a were treated with IFNγ (1,000 IU/ml) and IFNλ (50 ng/ml) as indicated. The immunoblotting was performed as indicated. (C) HepG2 cells were transfected with or without HA-USP2a, together with GAS-Luc and Renilla. The luciferase activity was measured 4 hrs after IFNγ (2,000 IU/ml) treatment. (D) HepG2 cells were transfected with or without Flag-USP2a, together with ISRE-Luc and Renilla. The luciferase activity was measured 4 hrs after IFNλ (50 ng/ml) treatment. (E, F) HepG2 cells transfected with shCON or shUSP2a were collected after IFNγ (1,000 IU/ml) or IFNλ (15 ng/ml) treatment for 6 hrs, and the mRNA levels of IFIT1 and Mx1 (E) or ISG15 and ISG54 (F) were analyzed by quantitative RT-PCR. (G) HepG2 cells transfected with empty vector or Flag-USP2a were treated with IFNγ (100 IU/ml) or IFNλ (0.5 ng/ml) overnight, and then cells were challenged by VSV-GFP (MOI = 0.5). After 24 hrs, VSV-GFP levels were detected by fluorescence. (H) KB cells were transfected with or without Flag-USP2a. KB cells were then challenged by VSV as indicated. pY701-STAT1 levels were determined by immunoblotting. (I) KB cells transfected with shCON and shUSP2a were infected with VSV for 2 hrs, the level of VSV viral RNA was analyzed by quantitative RT-PCR. (J) HUVEC cells transfected with shCON and shUSP2a were infected with DENV for 2 hrs, the level of DENV viral RNA was analyzed by quantitative RT-PCR. (K) A549 cells transfected with shCON and shUSP2a were infected with H1N1 for 2 hrs, the level of H1N1 viral RNA was analyzed by quantitative RT-PCR. *p<0.05, **p<0.01, ***p<0.001.

Given that pY701-STAT1 regulates all three classes of IFNs signaling, we next wanted to know whether USP2a enhanced type-II (IFNγ) and type-III (IFNλ) IFNs signaling and their corresponding antiviral functions. Consistent with the results obtained from our type-I IFN signaling experiments, knockdown of USP2a significantly attenuated pY701-STAT1 induced by IFNγ (Fig 7A) and IFNλ (Fig 7B). Overexpression of USP2a markedly enhanced GAS-Luciferase activity induced by IFNγ (Fig 7C), and ISRE-Luciferase activity induced by IFNλ (Fig 7D and S7D Fig). Likewise, knockdown of USP2a attenuated IFNγ-induced (Fig 7E) and IFNλ-induced (Fig 7F) ISGs expression. And overexpression of USP2a enhanced IFNγ-induced (S7E Fig) and IFNλ-induced (S7F Fig) ISGs expression. Moreover, USP2a overexpression promoted IFNγ- and IFNλ-mediated antiviral function as assessed by VSV-GFP replication in cells (Fig 7G).

Furthermore, we analyzed the effect of USP2a on the levels of infectious viral titers. Results showed that USP2a knockdown significantly inhibited IFNα-mediated (Fig 6J), as well as IFNγ- and IFNλ-mediated (S8B Fig) antiviral defense. Taken together, these results indicate that USP2a regulates pY701-STAT1 in all three classes of IFNs signaling and subsequently enhances their antiviral functions.

To analyze the effect of USP2a on antiviral signaling during an actual viral infection, human oral epidermoid KB cells were infected with VSV. We found that USP2a enhanced the level of antiviral signaling pY701-STAT1 during VSV infection (Fig 7H). Furthermore, the effect of USP2a on antiviral defense of KB cells was analyzed. We found that knockdown of USP2a significantly inhibited antiviral ability of KB cells against VSV (Fig 7I). In addition, we employed several other types of viruses, including dengue virus (DENV) and influenza A virus (H1N1, PR/8/34) to analyze the effect of USP2a on their pathophysiologically relevant cells. We found that knockdown of USP2a significantly inhibited antiviral ability of human vascular endothelial cells (HUVEC) against DENV (Fig 7J), and that of human lung cancer A549 cells against H1N1 (Fig 7K). These results suggest that USP2a plays important roles in antiviral defense of various types of cells.

## Discussion

STAT1, as a critical mediator of many cytokine signaling, becomes activated once phosphorylated at tyrosine 701 site (pY701-STAT1), and regulates diverse cellular processes including innate immune responses. Recently, several groups provided important evidence for the ubiquitination regulation of total STAT1 [12,13,17,18]. However, it seems that ubiquitination of total STAT1 is not sufficient to allow for a complete understanding of the regulation of cytokine signaling. As a matter of fact, total STAT1 protein levels are kept relatively stable even if protein synthesis has been inhibited for over 24 hours. Likewise, short treatment of cells with IFNs for a couple of hours does not obviously change the level of total STAT1 proteins. Therefore, these observations suggest that the early stages of IFNs-initiated STAT1 signaling may not be significantly affected by changes of total STAT1 ubiquitination and levels. Actually, STAT1-mediated signaling and function largely depend on activated STAT1 (pY701-STAT1). However, there is no evidence demonstrating whether the pY701-STAT1 form undergoes ubiquitination modification and subsequent regulation.

Here, our data clearly demonstrate that IFNα-induced pY701-STAT1 undergoes ubiquitination modification, and that the pY701-STAT1 form carries most of the STAT1 ubiquitination induced by IFNs signaling, when compared to the un-pY701-STAT1 forms (including pS727–STAT1) (Fig 1B). pY701-STAT1 is tightly regulated by proteasome-dependent degradation as demonstrated in Fig 1. This finding provides important evidence demonstrating that it is the pY701-STAT1 form—rather than other STAT1 forms—which largely induces STAT1 ubiquitination and regulates STAT1 levels by proteasome-dependent degradation in IFNs signaling. Importantly, although MG132 enhanced IFNs-induced pY701-STAT1 levels (Fig 1D), this experiment cannot exclude the possibility that MG132 inhibited the degradation of IFNs-activated upstream signaling (e.g. IFNs receptor, and Janus kinase JAK1/Tyk2), rather than that of pY701-STAT1. IFNs-stimulated ubiquitination and degradation of the IFNα receptor (IFNAR1) and K63-linked ubiquitination of Tyk2 have been previously reported [30,31,32]. Therefore, to provide more evidence that IFN-induced activated STAT1 can be regulated directly by proteasome degradation, we employed confocal immunofluorescence microscopy to observe the colocalization between IFN-induced activated STAT1 and the proteasome. Our data showed that IFNs induced nuclear import of STAT1, and nuclear pY701-STAT1 had clear colocalization with the nuclear proteasome (S3 Fig). This result supports our conclusion that IFNs-induced activated pY701-STAT1 can be regulated directly by proteasome-mediated degradation.

To provide more evidence for pY701-STAT1 ubiquitin-proteasome degradation, we inhibited the dephosphorylation of pY701-STAT1 (Fig 1G–1H, and S2B Fig). Previous studies on the negative regulation of pY701-STAT1 have long been focused on exploring dephosphorylation mechanisms [6,16]. Here, our data showed that blocking dephosphorylation of pY701-STAT1 could partially, but not completely, abolish the downregulation of pY701-STAT1(Fig 1G–1H, and S2B Fig). This suggests that pY701-STAT1 could also be regulated by other negative mechanisms. Furthermore, inhibition of proteasome-dependent degradation of pY701-STAT1 significantly blocked the downregulation of pY701-STAT1 (Fig 1I and S1D Fig). Hence, our data provided a new perspective that the ubiquitin-proteasome system plays an important role in the negative regulation of pY701-STAT1.

Interestingly, we found that ubiquitinated-pY701-STAT1 mainly accumulated in the nucleus (Fig 2A and 2B), and inhibition of nuclear import of pY701-STAT1 remarkably blocked pY701-STAT1 ubiquitination and downregulation (Fig 2C–2F). Hence, our finding suggests that pY701-STAT1 could be ubiquitinated and degraded in the nucleus. This finding is inconsistent with tons of other reports that describe ubiquitination-dependent degradation of cytoplasmic proteins and nuclear proteins by the cytoplasmic proteasome. However, several recent studies suggest that proteins in the nucleus could be degraded in the nucleus since proteasomal subunits have been shown to be enriched in the nucleus [33,34]. Gardner et al. reported that the turnover of unfolded and damaged nuclear proteins could occur in the nucleus [35]. However, recently, an inconsistent report showed that some nuclear proteins need to be exported from the nucleus and degraded in the cytoplasm [36]. Here, our results suggest that pY701-STAT1 could be largely degraded in the nucleus, which provides the direct evidence based on IFNs signaling for nuclear degradation of ubiquitinated proteins. However, further evidence is needed to completely address the question.

An important E3 ligase Smurf1 which targets STAT1 for ubiquitination has been identified, although it is currently unclear whether it mediates pY701-STAT1 ubiquitination, and in fact the interaction between Smurf1 and STAT1 is independent of IFNs treatment [17]. Another key nuclear E3 ligase SLIM can target both STAT1 and STAT4 for ubiquitination [37], which supports our understanding that STAT1 proteins can be regulated by ubiquitin-proteasome system in the nucleus. A recent report suggested that the deubiquitinase USP13 decreased STAT1 ubiquitination, which however was independent of IFNs treatment [18]. And USP13 was proved to mainly interact with nonactivated forms of STAT1 (un-pY701-STAT1) [18], which indicates that USP13 is not the deubiquitinase of pY701-STAT1. Here, we identified USP2a as an important deubiquitinase which regulates pY701-STAT1 ubiquitination and levels in the nucleus. USP2 family includes two major isoforms due to alternative splicing, USP2a and USP2b. USP2b has been demonstrated to cleave K63-linked ubiquitination of TBK1 and therefore inhibit IFNs production and antiviral activity [38]. Here, our data demonstrated that USP2a cleaves K48-linked ubiquitination of pY701-STAT1 and promotes IFNs signaling and antiviral function. Given that USP2a and USP2b are produced from the same USP2 transcript, it will be difficult to study the *in vivo* antiviral functions of USP2a using USP2-knockout mice model. This is because such a knockout could abolish both USP2a-mediated antiviral functions and USP2b-mediated proviral functions. To our knowledge, the work presented here uncovered the first deubiquitinase which regulates pY701-STAT1 ubiquitination in the nucleus. Moreover, the deubiquitinase USP2a enhances not only type-I but also type-II and type-III IFNs-mediated signaling and antiviral function.

**Fig 8:**
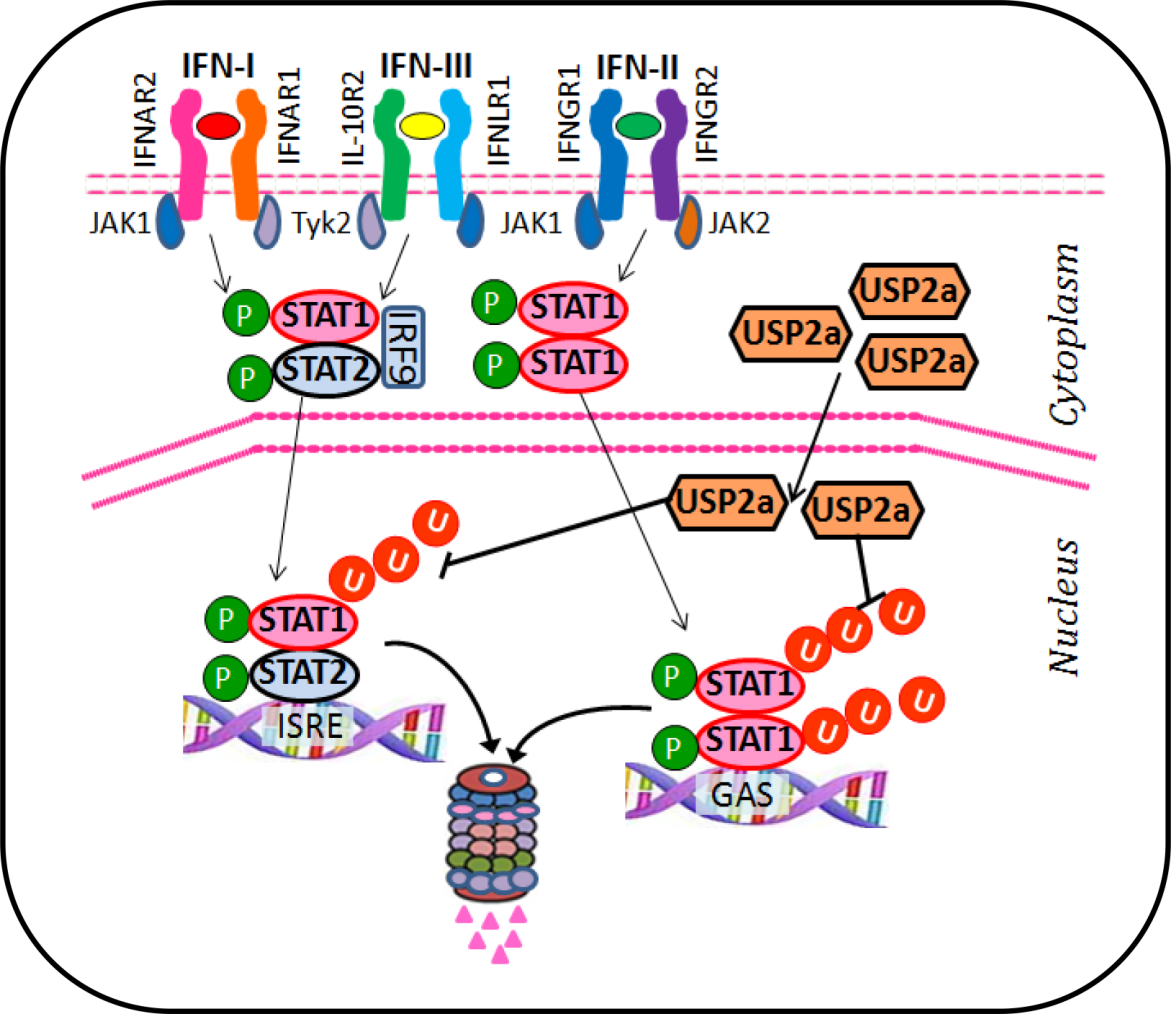
A proposed model of the regulation of pY701-STAT1 by ubiquitin-proteasome system. IFNs induce ubiquitination of pY701-STAT1. Ubiquitinated-pY701-STAT1 is mostly located in the nucleus and regulated by proteasome-dependent degradation. IFNs promote the nuclear import of the deubiquitinase USP2a, which binds to pY701-STAT1 and cleaves K48-linked ubiquitin chains of pY701-STAT1, and therefore sustains pY701-STAT1 levels in the nucleus. The positive regulation of pY701-STAT1 by USP2a enhances all three types of IFNs-induced signaling and antiviral function.

In summary, we elucidated that IFN-induced pY701-STAT1 largely raises STAT1 ubiquitination, which controls pY701-STAT1 levels in cells through proteasome-dependent degradation of pY701-STAT1. Ubiquitinated pY701-STAT1 is mostly located in the nucleus and could be largely regulated by the nuclear ubiquitin-proteasome system. We further demonstrated that type-I IFN signaling recruits USP2a to pY701-STAT1 and that USP2a stabilizes pY701-STAT1 by removing K48-linked ubiquitin chains. Finally we clarified that USP2a promotes all three types of IFNs-induced signaling and antiviral defenses (Fig 8). Further understanding of the biological significance of how USP2a regulates antiviral signaling could provide insight into enhancing IFNs therapeutic efficacy against viral infections.

## Materials and Methods

### Cell culture and transfection

293T, 2fTGH, A549, HT1080, HUVEC and HepG2 cell lines were obtained from ATCC. U3A cells [39] were kindly provided by Dr. GQ Chen. KB cells were obtained from the Cellbank of the Chinese Academy of Sciences (Shanghai, China). 293T, 2fTGH, A549, HT1080, U3A and HepG2 cells were cultured in Dulbecco’s modified Eagle’s medium (DMEM; HyClone). KB and HUVEC cells were cultured in Roswell Park Memorial Institute (RPMI) 1640 medium (HyClone). All medium were supplemented with 10% FBS (GIBCO, Life Technologies), 100 units/ml penicillin, and 100 μg/ml streptomycin. All cells were cultured at 37°C under 5% CO_2_. All transient transfections were carried out using Lipofectamine Plus (Invitrogen) or LongTrans (Ucallm) according to manufacturer’s instruction.

### Expression constructs and reagents

Flag-USP2a and catalytic inactive Flag-USP2a-C276A mutant were gifts from Dr. Simon S. Wing (McGill University). HA-USP2a and HA-USP2b were gifts from Dr. Chengjiang Gao (Shangdong University, China). Flag-STAT2 was a gift from Dr. Chunfu Zheng (Soochow University, China). 39 human USPs expression plasmids were gifts from Dr. J. Wade Harper (Harvard Medical School, Addgene plasmids). Flag-STAT1 was generated using PCR amplified from pIND-STAT1-V5 from Dr. Steven Johnson (Addgene # 11618). HA-Ub was a gift from Dr. Lingqiang Zhang (State Key Laboratory of Proteomics, China). ISRE-Luc, GAS-Luc and Renilla plasmids were gifts from Dr. Serge Y. Fuchs (University of Pennsylvania). shUSP2a, shKPNA1 and shImportin α plasmids were purchased from GENECHEM (Shanghai, China). All the mutations were generated by QuickChange site-Directed Mutagenesis Kit (Stratagene). All the plasmids were confirmed by DNA sequencing. Recombinant human IFNα, IFNγ and IFNλ were purchased from PBL Interferon Source. IFNα was used at the concentration of 1,000 IU/ml, unless stated otherwise. Cycloheximide, MG132, and other chemicals were purchased from Sigma. NSC87877 was from Santa Cruz (sc-204139).

### Immunoblotting

Cells were harvested using lysis buffer containing 150 mM NaCl, 20 mM Tris-HCl (PH7.4), 1% Nonidet P-40, 0.5 mM EDTA, PMSF (50 μg/ml) and protease inhibitors mixtures (Sigma). N-ethylmaleimide (10 mM) was added into the above lysis buffer when protein ubiquitination was detected. Equivalent protein quantities were subjected to SDS-PAGE or native-PAGE, and transferred to PVDF membranes. Membranes were then blocked with 5% non-fat milk or 5% BSA for 1 hr at room temperature and then probed with the primary antibodies, followed by the respective HRP-conjugated Goat anti-mouse or Goat anti-rabbit (Bioworld) secondary antibodies. Immunoreactive bands were visualized with SuperSignal West Dura Extended kits (Thermo Scientific). The following antibodies were used: antibodies against pY701-STAT1 (1:1,000, Cell Signaling, #9167), pS727-STAT1 (1:1000, cell signaling, #8826), pY690-STAT2 (1:1000, Santa Cruz, sc-21689-R), STAT1 (1:2,000, Cell Signaling, #9172), Ubiquitin (1:1,000, Santa Cruz, sc-8017), VSV-G (1:5,000, Santa Cruz, sc-66180), HA (1:2,000, abcam, ab9110), K48Ub (1:500, Cell Signaling, #4289), K63Ub (1:500, Millipore, #05–1308), USP2 (1:1,000, ABGENT, AP2131c), Flag (1:5,000, Sigma, F7425), Smurf1 (1:1000, Santa Cruz, sc-100616), β-Actin (1:5,000, Proteintech, #66009), KPNA1/Importin α5 (1:1,000, Santa Cruz, sc-101292), Lamin B1 (1:2,000, Proteintech, 12987-1-AP), Alpha-Tubulin (1:5,000, Proteintech, 66031-1-Ig). The Image J program (http://rsbweb.nih.gov/ij/download.html) was used for densitometric analysis of western blots.

### Immunoprecipitation

Cells were harvested in lysis buffer plus protease inhibitors mixture (Sigma) for 30 min on ice. After 12,000 g centrifugation for 15 min, the supernatant was collected and subjected to immunoprecipitation with specific antibody overnight on a rotor at 4°C. 20 μl Protein G agarose beads (Millipore, #16–266) were washed twice and then added and incubated for an additional 3 hrs on a rotor at 4°C. After washing five times with lysis buffer, the immunoprecipitates were eluted by boiling with loading buffer containing β–mercaptoethanol for 10 min and analysed by SDS-PAGE. For immunoprecipitation of Flag-tagged proteins, M2 Affinity Gel (Sigma, A2220) was used in lysates for 4 hrs on a rotor at 4°C. Then the immunoprecipitates were eluted and subjected to SDS-PAGE analysis as above.

### Cytoplasmic and nuclear proteins extraction

Cells were scraped in cold PBS-EDTA and then harvested in lysis buffer containing 10 mM HEPES (PH 7.9), 50 mM NaCl, 0.5 mM Sucrose, 0.1 mM EDTA, 0.5%Triton X-100, 1mM DTT, 10 mM Sodium pyrophosphate decahydrate, 0.5 M NaF, 0.2 M Na_3_VO_4_, 1 mM PMSF and protease inhibitors mixtures (Sigma), and then the supernatant was collected for the cytoplasmic extract after centrifuging for 10 min at 1,000 rpm. The pellet was resuspended with Buffer A containing 10 mM HEPES (PH 7.9), 10 mM KCl, 0.1 mM EGTA, 0.1 mM EDTA, 1 mM DTT, 1 mM PMSF and protease inhibitors mixtures. Centrifuge for 5 min at 1,000 rpm in swinging bucket rotor. Remove and discard supernatant. Add 4 volume of buffer C containing 10 mM HEPES (PH 7.9), 500 mM NaCl, 0.1 mM EGTA, 0.1 mM EDTA, 0.1% Nonidet P-40, 1 mM DTT, 1 mM PMSF and protease inhibitors mixtures. Vortex for 15 min at 4°C and then centrifuge for 10 min at 14,000rpm. Transfer the supernatant to get the nuclear extract. Tubulin-Alpha and Lamin B1 were used as loading control.

### Reporter gene assay

For analysis of IFNs-induced transcriptional activity, cells were transfected with the ISRE-Luciferase (or GAS-Luciferase) constructs together with Renilla plasmids and specific plasmids. After 36 hrs, cells were treated with IFNs overnight and then collected. For detection of IFNβ production, cells were transfected with the IFNβ-luciferase constructs together with Renilla plasmids and specific plasmids. After 36 hrs, cells were infected with virus for 20 hrs and then collected. The Luciferase activity was measured using the Dual-Luciferase Reporter Assay System (Promega, #E1910) according to the manufacturer's protocol. Activity was assayed in three independent experiments and is shown as the average mean ± standard deviation (s.d.).

### In vivo deubiquitination assay

Cells were transfected with Flag-USP2a or shUSP2a, together with or without HA-ubiquitin. 48 hrs after transfection, cells were treated with or without IFNα (1,000 IU/ml) for 15 min, and then harvested in lysis buffer containing 150 mM NaCl, 20 mM Tris-HCl (PH 7.4), 1% Nonidet P-40, 0.5 mM EDTA, PMSF (50 μg/ml), N-ethylmaleimide (10 mM) and protease inhibitors mixtures (Sigma). For analysis of the deubiquitination effect of USP2a on IFNα-induced STAT1 ubiquitination, STAT1 proteins were immunoprecipitated using specific STAT1 antibody and then subjected to ubiquitination analysis using HA antibody by western blotting. For analysis of the deubiquitination effect of USP2a on IFNα-induced pY701-STAT1 ubiquitination, pY701-STAT1 proteins were immunoprecipitated using specific pY701-STAT1 antibody and then subjected to ubiquitination analysis using ubiquitin or K48Ub or K63Ub specific antibody by western blotting.

### Cycloheximide chase assay

The half-life of pY701-STAT1 or total STAT1 was determined by cycloheximide (CHX) chase assay. For analysis of pY701-STAT1 levels, 293T cells were seeded in 6-well cell culture plates and were transfected with Flag-STAT1. 48 hrs after transfection, cells were treated with DMSO or CHX (50 μg/ml) for indicated time points. Cells were then collected and subjected to analysis by western blotting. For analysis of total STAT1 levels, 293T cells were transfected with control shRNA or USP2a shRNA. 48 hrs after transfection, cells were treated with DMSO or CHX (50 μg/ml) for indicated time points, and then the equal amount of boiled lysates were analysed by western blotting.

### RNA isolation and real-time PCR

Total RNAs were isolated from 293T cells using Trizol reagent (Invitrogen). cDNA was synthesized by reverse transcription using oligo (dT) and subjected to quantitative real-time PCR with IFIT1, ISG15, ISG54 and β-ctin primers in the presence of SYBR Green Supermix (BIO-RAD). The primer sequences are as following:

IFIT1 (5'-CACAAGCCATTTTCTTTGCT-3' and 5'-ACTTGGCTGCATATCGAAAG-3'); ISG15 (5'-GGGACCTGACGGTGAAGATG-3' and 5'-CGCCGATCTTCTGGGTGA T-3'); ISG54 (5'-CACCTCTGGACTGGCAATAGC-3' and 5'-GTCAGGATTCAGCCG AATGG-3'); Mx1 (5'-ACATCCAGAGGCAGGAGACAATC-3' and 5'-TCCACCAGATC AGGCTTCGTCAA-3'); VSV (5’-ACGGCGTACTTCCAGATGG-3’ and 5’-CTCGGTTC AAGATCCAGGT-3’); H1N1 (5’-TTCTAACCGAGGTCGAAACG-3’ and 5’-ACAAAGC GTCTACGCTGCAG-3’); DENV (5’-CATTCCAAGTGAGAATCTCTTTGTCA-3’ and 5’-CAGATCTCTGATGAATAACCAACG-3’); β-actin (5'-ACCAACTGGGACGACAT GGAGAAA-3' and 5'-ATAGCACAGCCTGGATAGCAACG-3').

The relative expression of the target genes was normalized to β-actin mRNA. The results were analyzed from three independent experiments and shown as the average mean ± standard deviation (s.d.).

### Flow cytometry analysis and immunofluorescence microscopy

293T cells infected with VSV-GFP (MOI = 0.5) were subjected to analysis by immunofluorescence microscopy directly or by flow cytometry. Briefly, for immunofluorescence microscopy analysis, VSV-GFP viruses were pictured with upright fluorescence microscope (Tokyo, Japan). Magnification was ×200. For flow cytometry analysis, cells were collected with cold 1 × PBS and were acquired in a FACS Calibur (BD Biosciences) equipped with a 488 nm argon laser and a 635 nm red diode laser. Data were analyzed with FlowJo Software (FlowJo, Ashland, OR, USA). For colocalization analysis, HeLa cells were treated with IFNα for 60 min and fixed in 100% Methanol at -20°C, permeabilized with 0.5% Triton X100 and blocked with 1%BSA. Sample were incubated overnight with both an anti-pY701-STAT1 antibody (abcam, ab29045) and an anti-PA28β antibody (Cell Signaling, 2409) in 0.5%BSA.After three washes with PBS, cells were stained with 488 goat anti-mouse IgG (Alexa Fluor, A11001) or 594 goat anti-rabbit IgG (Alexa Fluor, A11012). Cell nuclei were stained with DAPI. The fluorescent images were captured with the Nikon A1 confocal microscope. Manders overlap coefficient (R) was used for measurement of the extent of colocalization in the image. R values indicating colocalization: from 0.6 to 1.0 [40].

### DUB screening

Human USPs expression plasmids were from Dr. J. Wade Harper (Harvard Medical School, Addgene plasmids). 293T cells seeded in 24-well plates were transfected with each USP. 48 hrs after trasnfection, cells were treated with 1,000 IU/ml IFNα for 30 min. Cells were harvested at once and the level of pY701-STAT1 and β-actin were detected by western blot using specific antibody. USP2a was selected for further study based on the best induction fold as compared with control vector transfection.

### Virus and viral infection

Vesicular stomatitis virus (VSV) and Sendai virus (SeV) were gifts from Dr. Chen Wang (Shanghai Institutes for Biological Sciences, Chinese Academy of Sciences, China). Dengue virus (DENV) and Influenza A virus (H1N1, PR/8/34) were from Dr. Jianfeng Dai (Institutes of Biology and Medical Sciences, Soochow University). VSV-GFP was a gift from Dr. Chunsheng Dong (Soochow University, China). The antiviral effect of IFNα was determined by pretreating cells overnight prior to infection with viruses. Briefly, cells were transfected with Flag-USP2a or shUSP2a. 48 hrs after transfection, cells were pretreated with IFNα (50 IU/ml) overnight. After washing twice, cells were infected with VSV or VSV-GFP at a multiplicity of infection (MOI) of 1.0 or 0.5 for 1.5 hrs. Then the infection medium was removed by washing twice. Cells were fed with fresh medium and incubated for 20 hrs. Cells were analysed by immunofluorescence or FACS or western blotting using VSV-G antibody. To assess the antiviral ability of cells (KB, HUVEC, and A549) against their corresponding viruses, shControl or shUSP2a plasmids were transfected into cells. 48 hrs after transfection, cells were challenged with VSV, or DENV, or H1N1 for 2 hrs. Then cells were collected and viral RNAs were analyzed by Real Time quantitative PCR. For analysis of infectious viral titers, cell culture supernatant after virus infection was collected. Serial dilutions of viral supernatant were cultured on confluent layers of HeLa cells overlaid with 0.5% agarose gels prepared in DMEM and 10% FBS culture medium. After 48–72 hrs post infection, HeLa cells were washed and fixed by 4% formalin for 10 min, and then were stained by crystal violet. The plaque-forming units (PFU/ml) were calculated.

### Statistical analysis

Comparison between different groups was analyzed using Student’s T-test. Data represent the mean ± SEM. All differences were considered statistically significant with p < 0.05.

## Supporting Information

S1 Fig Phosphorylation and ubiquitination of Flag-STAT1. (A) 293T cells transfected with or without Flag-STAT1-WT/YF as indicated were treated with IFNα for 15 min. Flag-STAT1 proteins were immunoprecipitated by Flag antibody, and ubiquitination of Flag-STAT1 was detected by ubiquitin antibody. (B) Whole cell lysates from untransfected 293T cells or 293T cells transfected with Flag-STAT1 were subjected to immunoprecipitation using STAT1 antibody or Flag antibody. pY701-STAT1 levels were detected by immunoblotting as indicated. (C) 293T cells were transfected with Flag-STAT1-WT/KKRR. The cytoplasm and the nucleus were separated and used to determine the level of pY701-Flag-STAT1 and total Flag-STAT1 by immunobloting. (D) 293T cells transfected with Flag-STAT1 were pretreated with or without MG132 (50 μM) for 2 hrs, and then treated with IFNα (1,000 IU/ml) for 15 min. IFNα was removed by washing twice. Cells were further incubated using DMEM with or without MG132 (50 μM) as indicated times. pY701-STAT1-Flag and total Flag-STAT1 were analyzed as indicated. Quantification of pY701 -STAT1-Flag protein levels were analyzed by normalization to total Flag-STAT1.
(TIF)

S2 Fig Downregulation of pY701—STAT1. (A) 293T cells transfected with control shRNA (shCON) or USP2a shRNA (shUSP2a) were treated with CHX as indicated. The immunoblotting was performed as indicated. (B) 293T cells pretreated with NSC87877 for 3 hrs were stimulated with IFNα for 15 min. Cells were further incubated after IFNα removal. pY701-STAT1 proteins were analyzed as indicated. (C) 293T cells transfected with HA-Ub were stimulated with IFNα for 15 min. The proteins from the cytoplasm and the nucleus were analyzed using indicated antibodies. (D) 293T cells transfected with shCON or shSmurf1 were treated with IFNα for 15 min. pY701-STAT1 proteins were immunoprecipitated, and immunoblotting was performed as indicated.
(TIF)

S3 Fig Colocolization of IFNα-induced nuclear pY701-STAT1 and the proteasome in the nucleus. HeLa cells were treated with IFNα (1,000 IU/ml) for 60 min and then were stained by a pY701-STAT1 antibody and a PA28β antibody. Cell nuclei were stained with DAPI. The fluorescent images were captured with the Nikon A1 confocal microscope. Manders overlap coefficient (R*, values indicating colocalization: from 0.6 to 1.0) [40] was used for the measurement of the extent of colocalization between pY701-STAT1 and PA28β.
(TIF)

S4 Fig Identification of USP2a as the deubiquitinase for pY701-STAT1 regulation. (A) 293T cells transfected with empty vector or HA-USP2a or Flag-HA-USP30, or Flag-HA-USP52 were treated with IFNα for 15 min. pY701-STAT1 proteins were immunoprecipitated by pY701 antibody. The immunoblotting was performed as indicated. (B) 293T cells were transfected with increased amount of HA-USP2a. TC-PTP levels were analyzed as indicated. (C) 293T cells transfected with shRNAs against Importin α were treated with IFNα for 30 min. The levels of nuclear USP2a were analyzed by immunoblotting. (D) 293T cells transfected with Flag-STAT1-KKRR and shUSP2a were treated with IFNα for 15 min, then IFNα was removed. Cells were further incubated as indicated times. Flag-STAT1 proteins were immunoprecipitated by Flag antibody, and levels of pY701-STAT1-KKRR were analyzed as indicated. (E) 293T cells were transfected with Flag-STAT1-WT or -YF. Flag-STAT1-WT/YF was immunoprecipitated, and endogenous USP2a was detected as indicated. (F) 293T cells were stimulated with IFNα (1,000 IU/ml) for 15 min. pY701-STAT1 proteins were separated by immunoprecipitated using pY701-STAT1 antibody. The supernatant from pY701-STAT1-immunoprecipitation was subjected to STAT1 immunoprecipitation using STAT1 antibody. The levels of USP2a and STAT1 were analyzed as indicated. (G) 293T cells transfected with or without Flag-USP2a were treated with IFNα for 15 min. pY701-STAT1 or pS727-STAT1 proteins were immunoprecipitated, and then Flag-USP2a was detected as indicated.
(TIF)

S5 Fig Detection of human USP2a and USP2b using USP2 antibody. (A) 293T cells transfected with Flag-USP2a or Flag-USP2b were subjected to immunoblotting using USP2 antibody. (B) The expression of USP2a and USP2b in different cell lines was detected by USP2 antibody. (C) 293T cells treated with IFNα for indicated times. The levels of USP2a and USP2b were detected as indicated.
(TIF)

S6 Fig USP2a deubiquitinates pY701-STAT1. (A) 293T cells transfected with empty vector or Flag-USP2a were treated with or without IFNα (1,000 IU/ml) as indicated. STAT1 proteins were immunoprecipitated and the immunoblotting was performed as indicated. (B) 293T cells transfected with or without Flag-USP2a were treated with IFNα for 15 min. pY701-STAT1 proteins were immunoprecipitated by pY701 antibody, and immunoblotting was performed as indicated. (C) 293T cells transfected with Flag-STAT2, together with empty vector or HA-USP2a, were treated with or without IFNα (1,000 IU/ml) as indicated. Flag-STAT2 proteins were immunoprecipitated and the immunoblotting was performed as indicated. (D) 293T cells transfected with Flag-STAT2, together with empty vector or shUSP2a, were treated with or without IFNα (1,000 IU/ml) as indicated. Flag-STAT2 proteins were analyzed as (C). (E) 293T cells transfected with or without Flag-USP2a were treated with IFNα (500 IU/ml) as indicated. pY701-STAT1 levels are analyzed by immunoblotting. (F) 293T cells transfected with Flag-USP2a were treated with IFNα for 30 min. The nuclear proteins were separated, and then were subjected to native-PAGE analysis by indicated antibodies.
(TIF)

S7 Fig USP2a enhances IFNs induced signaling. (A) 293T cells transfected with empty vector or Flag-USP2a were treated with IFNα for 24 hrs, and then STAT1, Flag-USP2a and β-actin were immunoblotted using indicated antibodies. (B) 293T cells transfected with or without Flag-USP2a-WT/C276A were infected with VSV. After 20 hrs cells were collected and VSV-G proteins were detected by immunoblotting. (C) 293T cells were transfected with empty vector or Flag-USP2a, together with IFNβ-Luc and Renilla. The IFNβ-Luc activity was analyzed after infection with SeV for 20 hrs. (D) HepG2 cells were transfected with or without HA-USP2a, together with ISRE-Luc and Renilla. The luciferase activity was measured 4 hrs after IFNλ (50 ng/ml) treatment. *p<0.05, **p<0.01. (E, F) HepG2 cells transfected with or without Flag-USP2a were collected after IFNγ (1,000 IU/ml) or IFNλ (15 ng/ml) treatment for 6 hrs, and the mRNA levels of IFIT1 and Mx1 (E) or ISG15 and ISG54 (F) were analyzed by quantitative RT-PCR.
(TIF)

S8 Fig USP2a enhances IFNs induced antiviral defense. (A) 2fTGH and U3A cells transfected with or without HA-USP2a were infected with VSV (MOI = 1.0). After 20 hrs, cells were collected and immunoblotting was performed as indicated. (B) 293T cells transfected with shCON or shUSP2a were treated with IFNγ (100 IU/ml) or IFNλ (0.5 ng/ml) overnight, and then cells were challenged by VSV-GFP (MOI = 0.5). After 24 hrs, cell culture supernatant was collected, and a plaque assay was used for analysis of infectious viral titers. *p<0.05, **p<0.01, ***p<0.001.
(TIF)

S1 Table List of ID numbers for genes and proteins in this study. (DOCX)
