# Supplementary figures and images for: Deubiquitinase USP2a Sustains Interferons Antiviral Activity by Restricting Ubiquitination of Activated STAT1 in the Nucleus
Source: PLoS Pathog. 2016 Jul 19;12(7):e1005764. doi: 10.1371/journal.ppat.1005764 (PMC4951015; doi:10.1371/journal.ppat.1005764)

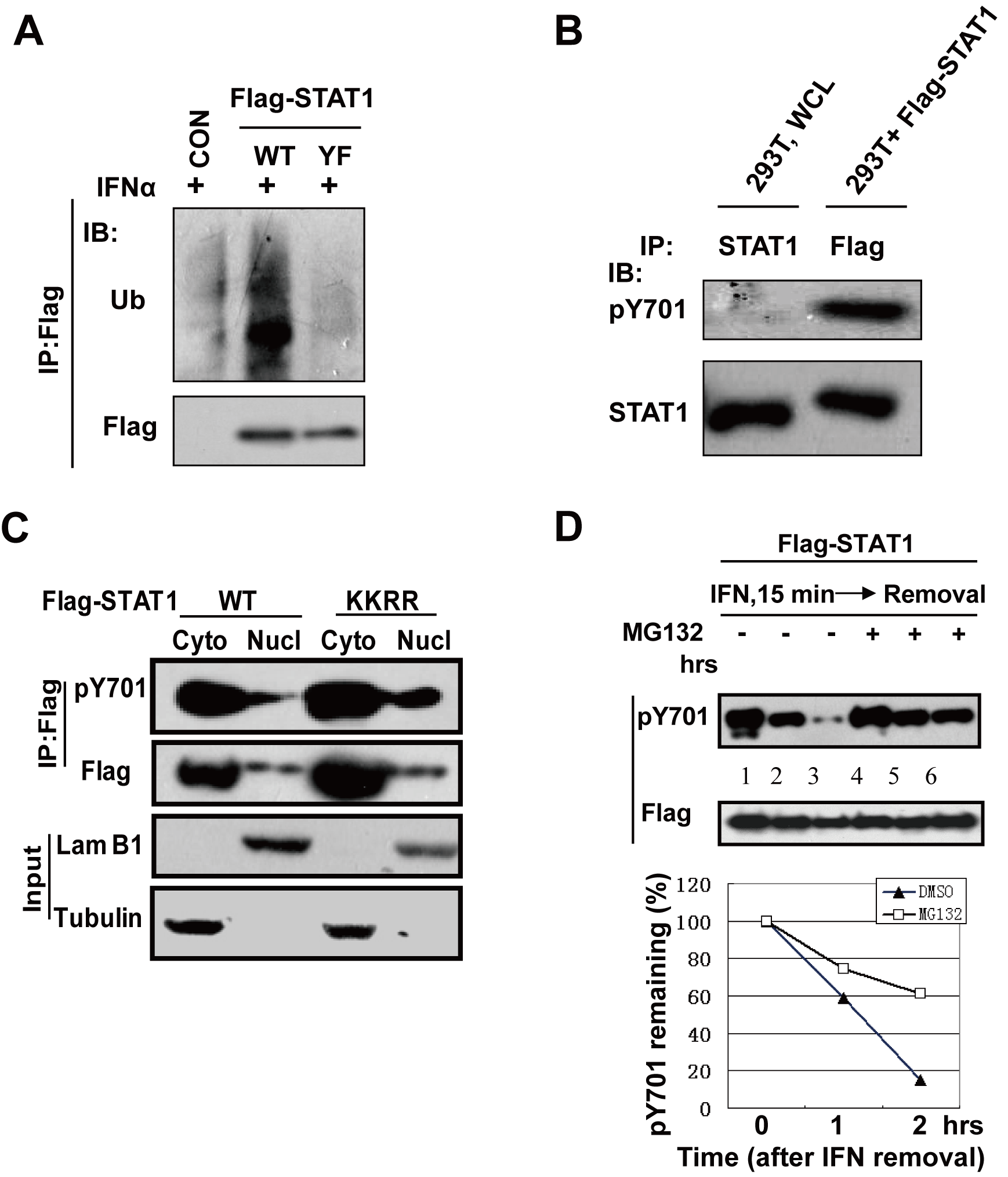

Supplement: S1 Fig — (A) 293T cells transfected with or without Flag-STAT1-WT/YF as indicated were treated with IFNα for 15 min. Flag-STAT1 proteins were immunoprecipitated by Flag antibody, and ubiquitination of Flag-STAT1 was detected by ubiquitin antibody. (B) Whole cell lysates from untransfected 293T cells or 293T cells transfected with Flag-STAT1 were subjected to immunoprecipitation using STAT1 antibody or Flag antibody. pY701-STAT1 levels were detected by immunoblotting as indicated. (C) 293T cells were transfected with Flag-STAT1-WT/KKRR. The cytoplasm and the nucleus were separated and used to determine the level of pY701-Flag-STAT1 and total Flag-STAT1 by immunobloting. (D) 293T cells transfected with Flag-STAT1 were pretreated with or without MG132 (50 μM) for 2 hrs, and then treated with IFNα (1,000 IU/ml) for 15 min. IFNα was removed by washing twice. Cells were further incubated using DMEM with or without MG132 (50 μM) as indicated times. pY701-STAT1-Flag and total Flag-STAT1 were analyzed as indicated. Quantification of pY701 -STAT1-Flag protein levels were analyzed by normalization to total Flag-STAT1. (TIF) [file ppat.1005764.s001.tif]

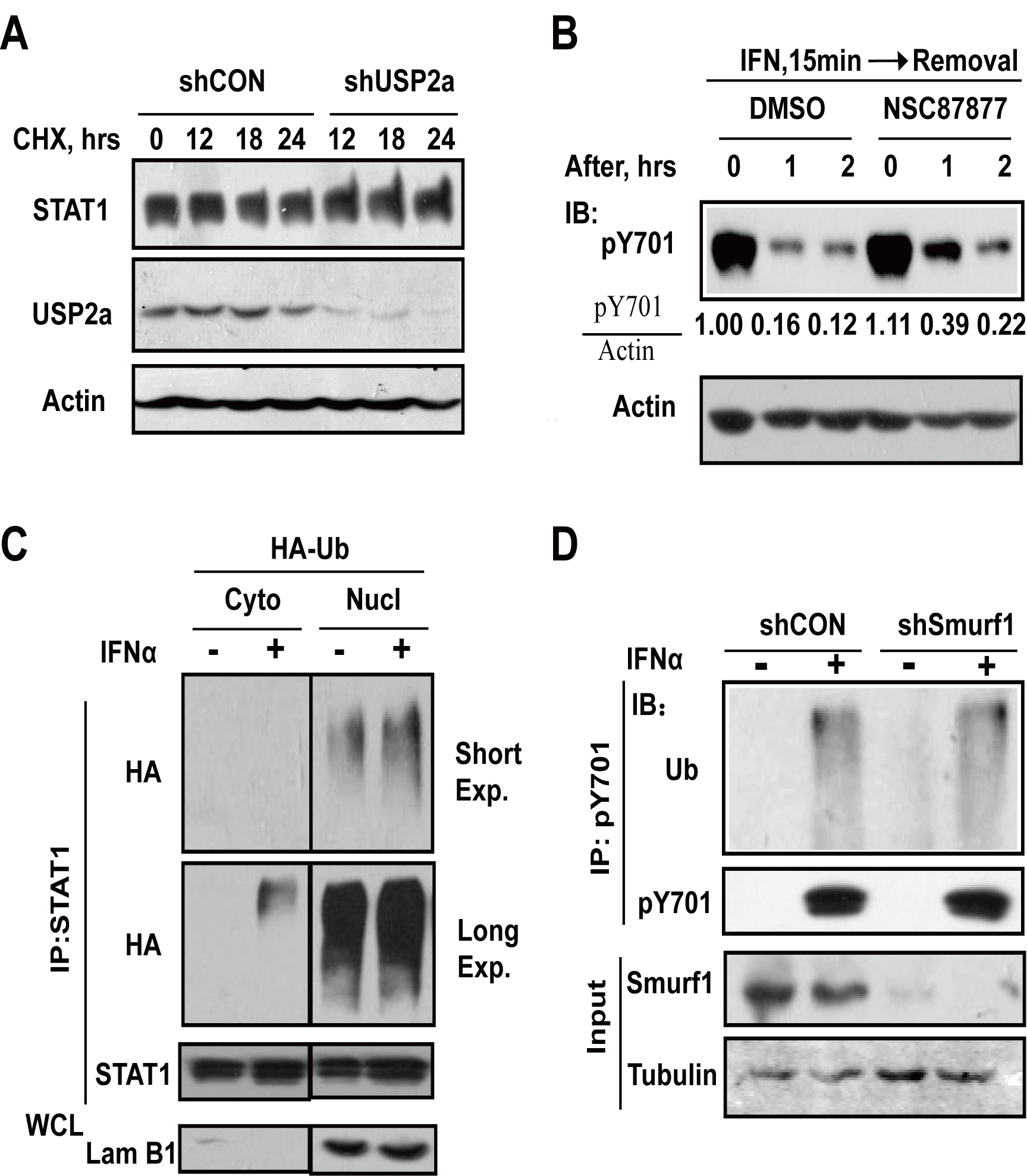

Supplement: S2 Fig — (A) 293T cells transfected with control shRNA (shCON) or USP2a shRNA (shUSP2a) were treated with CHX as indicated. The immunoblotting was performed as indicated. (B) 293T cells pretreated with NSC87877 for 3 hrs were stimulated with IFNα for 15 min. Cells were further incubated after IFNα removal. pY701-STAT1 proteins were analyzed as indicated. (C) 293T cells transfected with HA-Ub were stimulated with IFNα for 15 min. The proteins from the cytoplasm and the nucleus were analyzed using indicated antibodies. (D) 293T cells transfected with shCON or shSmurf1 were treated with IFNα for 15 min. pY701-STAT1 proteins were immunoprecipitated, and immunoblotting was performed as indicated. (TIF) [file ppat.1005764.s002.tif]

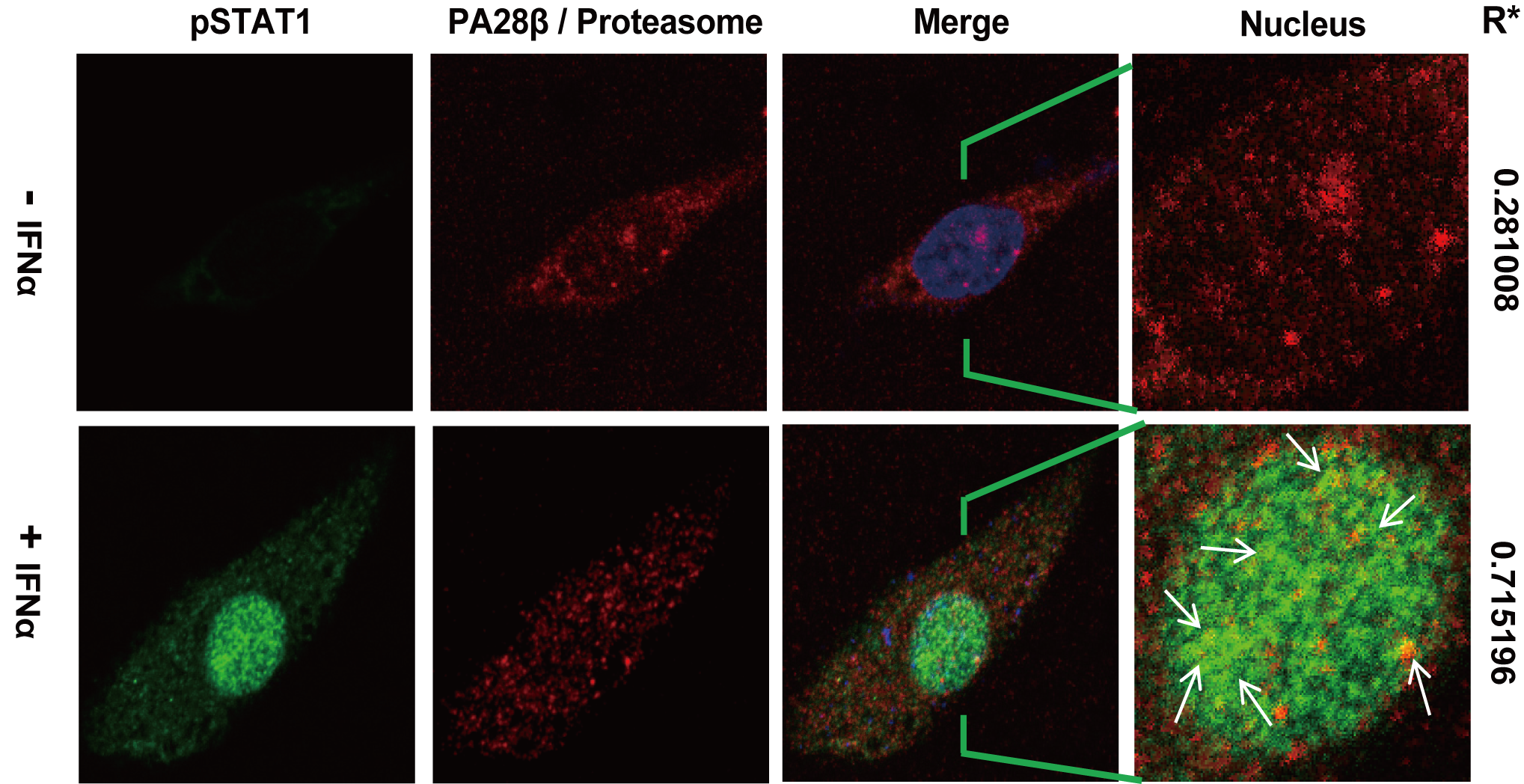

Supplement: S3 Fig — HeLa cells were treated with IFNα (1,000 IU/ml) for 60 min and then were stained by a pY701-STAT1 antibody and a PA28β antibody. Cell nuclei were stained with DAPI. The fluorescent images were captured with the Nikon A1 confocal microscope. Manders overlap coefficient (R*, values indicating colocalization: from 0.6 to 1.0) [40] was used for the measurement of the extent of colocalization between pY701-STAT1 and PA28β. (TIF) [file ppat.1005764.s003.tif]

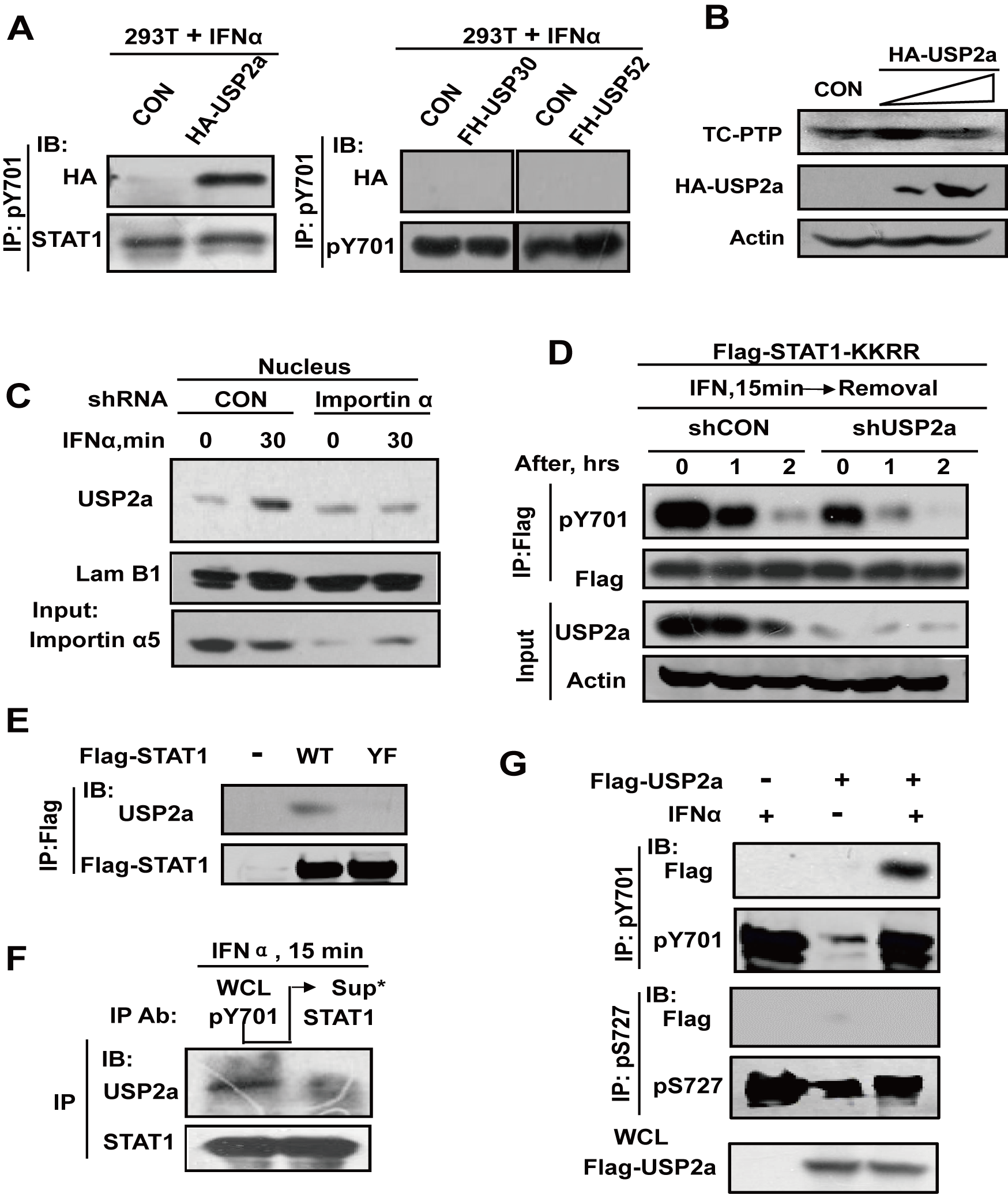

Supplement: S4 Fig — (A) 293T cells transfected with empty vector or HA-USP2a or Flag-HA-USP30, or Flag-HA-USP52 were treated with IFNα for 15 min. pY701-STAT1 proteins were immunoprecipitated by pY701 antibody. The immunoblotting was performed as indicated. (B) 293T cells were transfected with increased amount of HA-USP2a. TC-PTP levels were analyzed as indicated. (C) 293T cells transfected with shRNAs against Importin α were treated with IFNα for 30 min. The levels of nuclear USP2a were analyzed by immunoblotting. (D) 293T cells transfected with Flag-STAT1-KKRR and shUSP2a were treated with IFNα for 15 min, then IFNα was removed. Cells were further incubated as indicated times. Flag-STAT1 proteins were immunoprecipitated by Flag antibody, and levels of pY701-STAT1-KKRR were analyzed as indicated. (E) 293T cells were transfected with Flag-STAT1-WT or -YF. Flag-STAT1-WT/YF was immunoprecipitated, and endogenous USP2a was detected as indicated. (F) 293T cells were stimulated with IFNα (1,000 IU/ml) for 15 min. pY701-STAT1 proteins were separated by immunoprecipitated using pY701-STAT1 antibody. The supernatant from pY701-STAT1-immunoprecipitation was subjected to STAT1 immunoprecipitation using STAT1 antibody. The levels of USP2a and STAT1 were analyzed as indicated. (G) 293T cells transfected with or without Flag-USP2a were treated with IFNα for 15 min. pY701-STAT1 or pS727-STAT1 proteins were immunoprecipitated, and then Flag-USP2a was detected as indicated. (TIF) [file ppat.1005764.s004.tif]

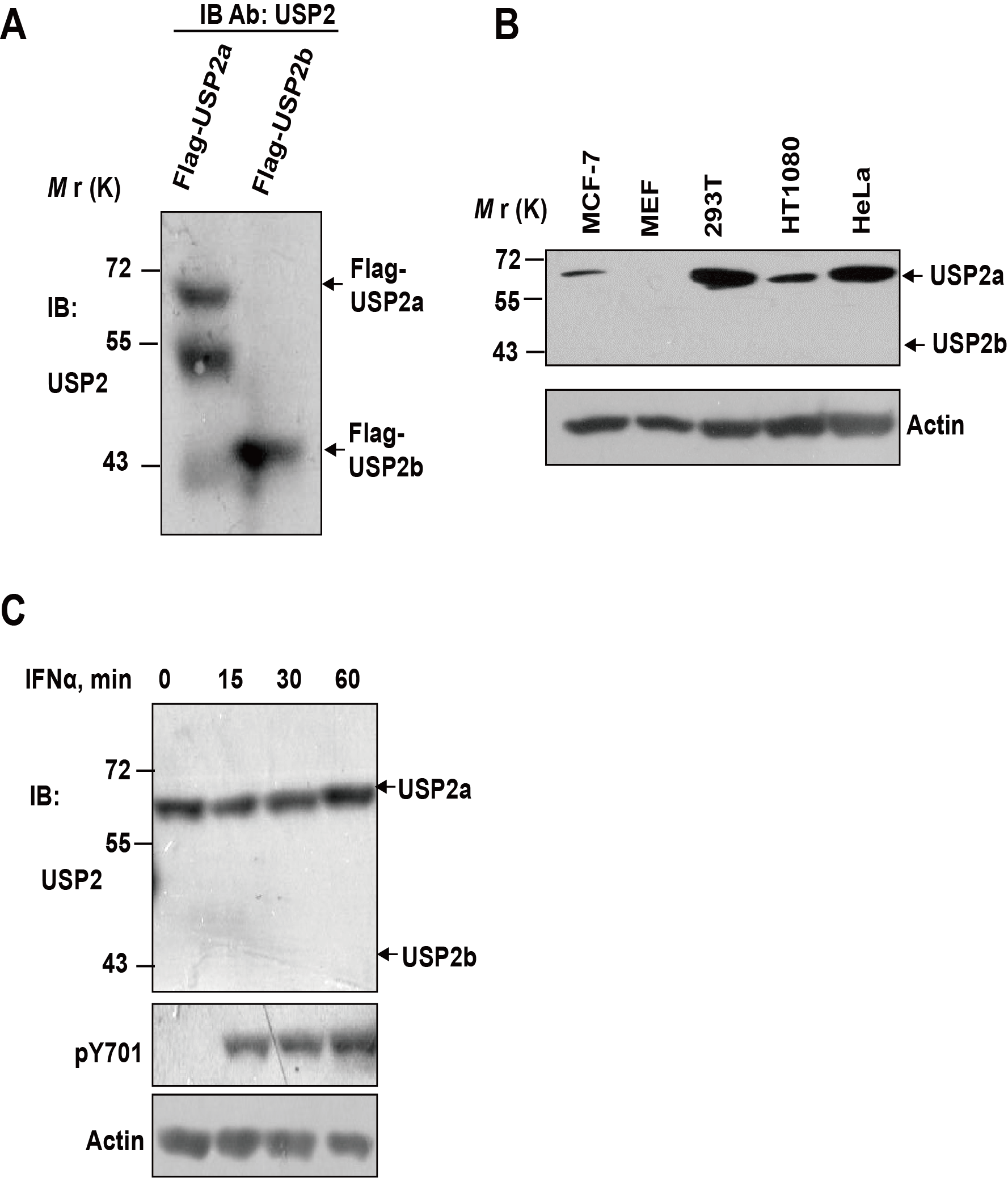

Supplement: S5 Fig — (A) 293T cells transfected with Flag-USP2a or Flag-USP2b were subjected to immunoblotting using USP2 antibody. (B) The expression of USP2a and USP2b in different cell lines was detected by USP2 antibody. (C) 293T cells treated with IFNα for indicated times. The levels of USP2a and USP2b were detected as indicated. (TIF) [file ppat.1005764.s005.tif]

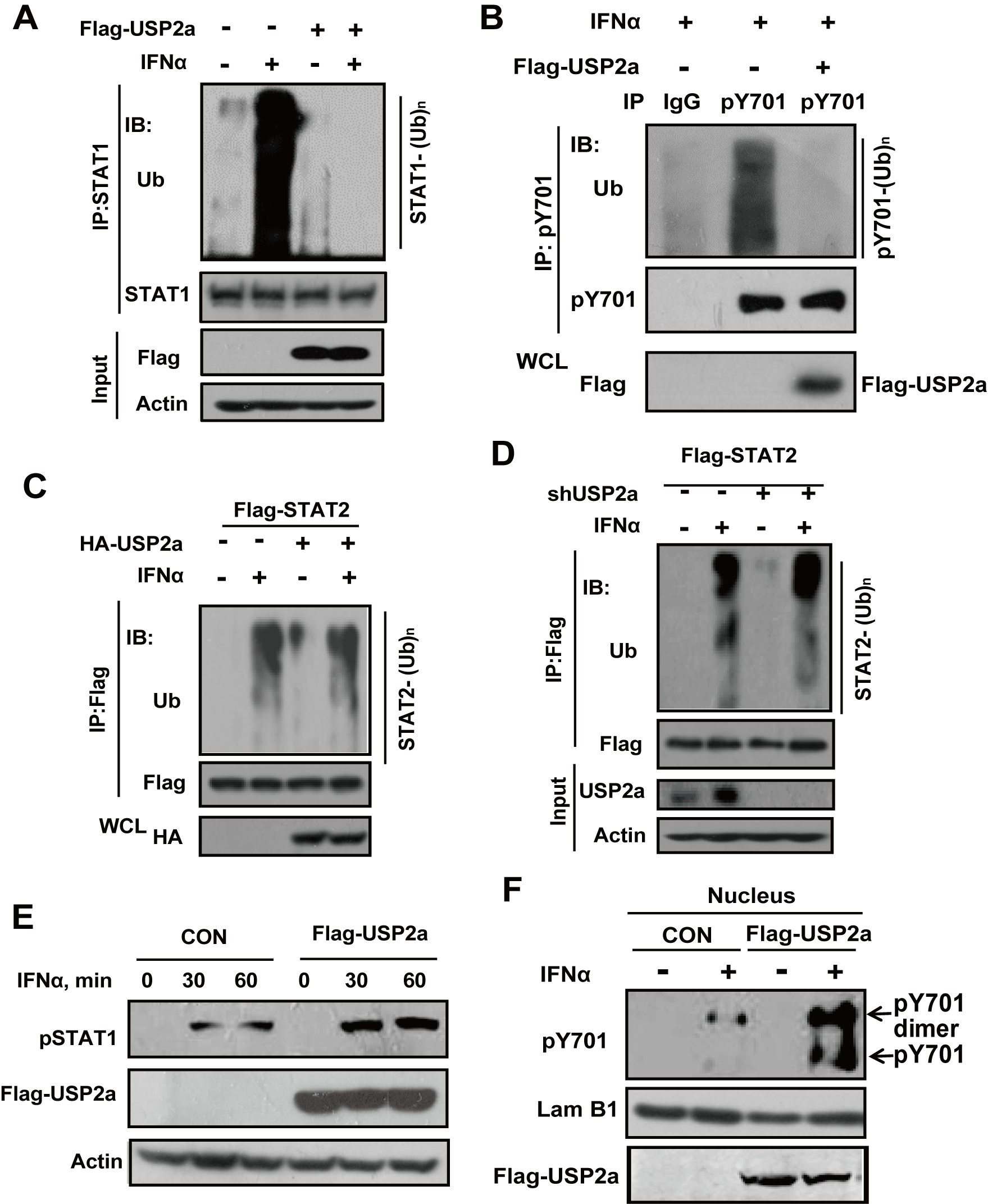

Supplement: S6 Fig — (A) 293T cells transfected with empty vector or Flag-USP2a were treated with or without IFNα (1,000 IU/ml) as indicated. STAT1 proteins were immunoprecipitated and the immunoblotting was performed as indicated. (B) 293T cells transfected with or without Flag-USP2a were treated with IFNα for 15 min. pY701-STAT1 proteins were immunoprecipitated by pY701 antibody, and immunoblotting was performed as indicated. (C) 293T cells transfected with Flag-STAT2, together with empty vector or HA-USP2a, were treated with or without IFNα (1,000 IU/ml) as indicated. Flag-STAT2 proteins were immunoprecipitated and the immunoblotting was performed as indicated. (D) 293T cells transfected with Flag-STAT2, together with empty vector or shUSP2a, were treated with or without IFNα (1,000 IU/ml) as indicated. Flag-STAT2 proteins were analyzed as (C). (E) 293T cells transfected with or without Flag-USP2a were treated with IFNα (500 IU/ml) as indicated. pY701-STAT1 levels are analyzed by immunoblotting. (F) 293T cells transfected with Flag-USP2a were treated with IFNα for 30 min. The nuclear proteins were separated, and then were subjected to native-PAGE analysis by indicated antibodies. (TIF) [file ppat.1005764.s006.tif]

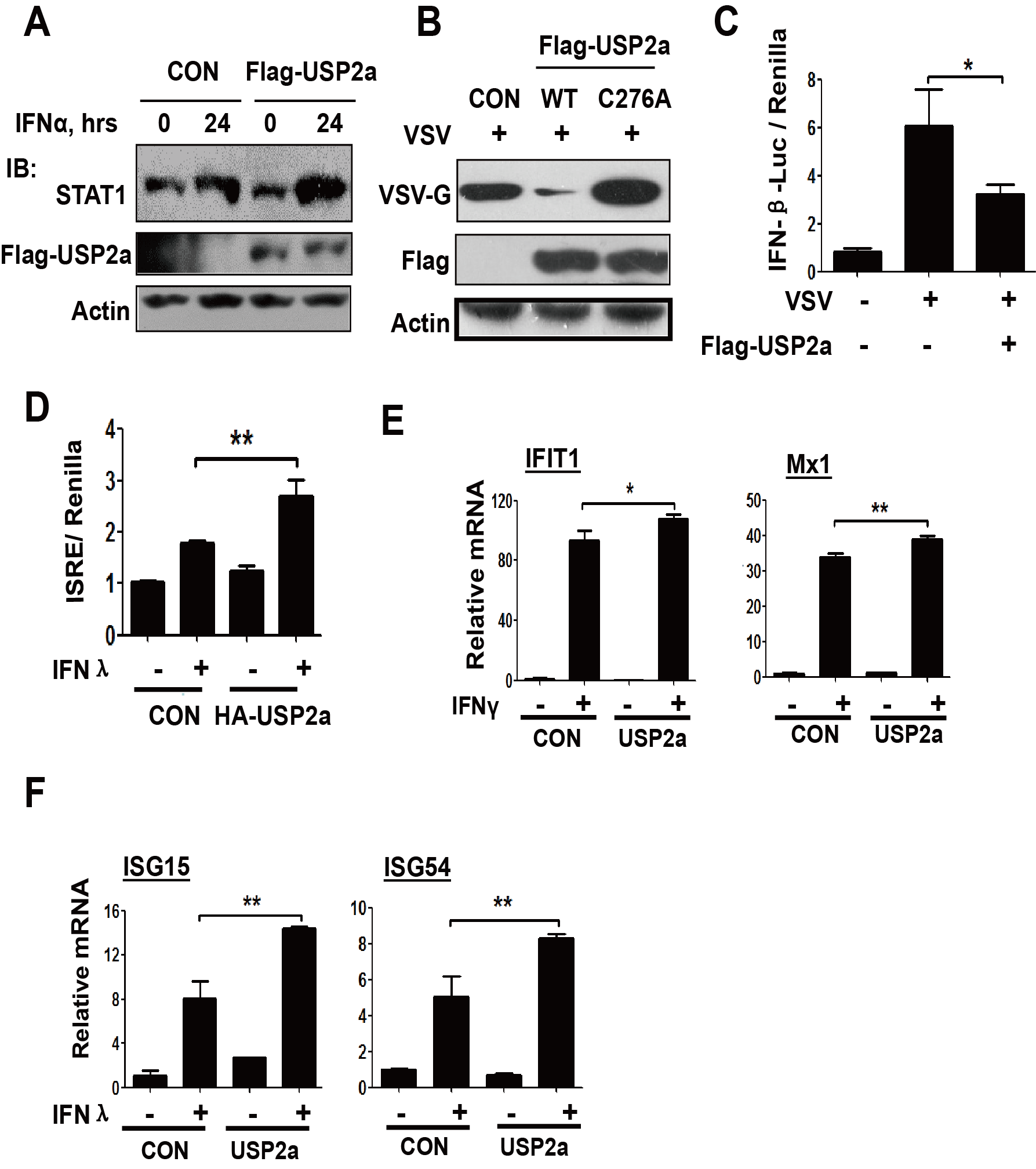

Supplement: S7 Fig — (A) 293T cells transfected with empty vector or Flag-USP2a were treated with IFNα for 24 hrs, and then STAT1, Flag-USP2a and β-actin were immunoblotted using indicated antibodies. (B) 293T cells transfected with or without Flag-USP2a-WT/C276A were infected with VSV. After 20 hrs cells were collected and VSV-G proteins were detected by immunoblotting. (C) 293T cells were transfected with empty vector or Flag-USP2a, together with IFNβ-Luc and Renilla. The IFNβ-Luc activity was analyzed after infection with SeV for 20 hrs. (D) HepG2 cells were transfected with or without HA-USP2a, together with ISRE-Luc and Renilla. The luciferase activity was measured 4 hrs after IFNλ (50 ng/ml) treatment. *p<0.05, **p<0.01. (E, F) HepG2 cells transfected with or without Flag-USP2a were collected after IFNγ (1,000 IU/ml) or IFNλ (15 ng/ml) treatment for 6 hrs, and the mRNA levels of IFIT1 and Mx1 (E) or ISG15 and ISG54 (F) were analyzed by quantitative RT-PCR. (TIF) [file ppat.1005764.s007.tif]

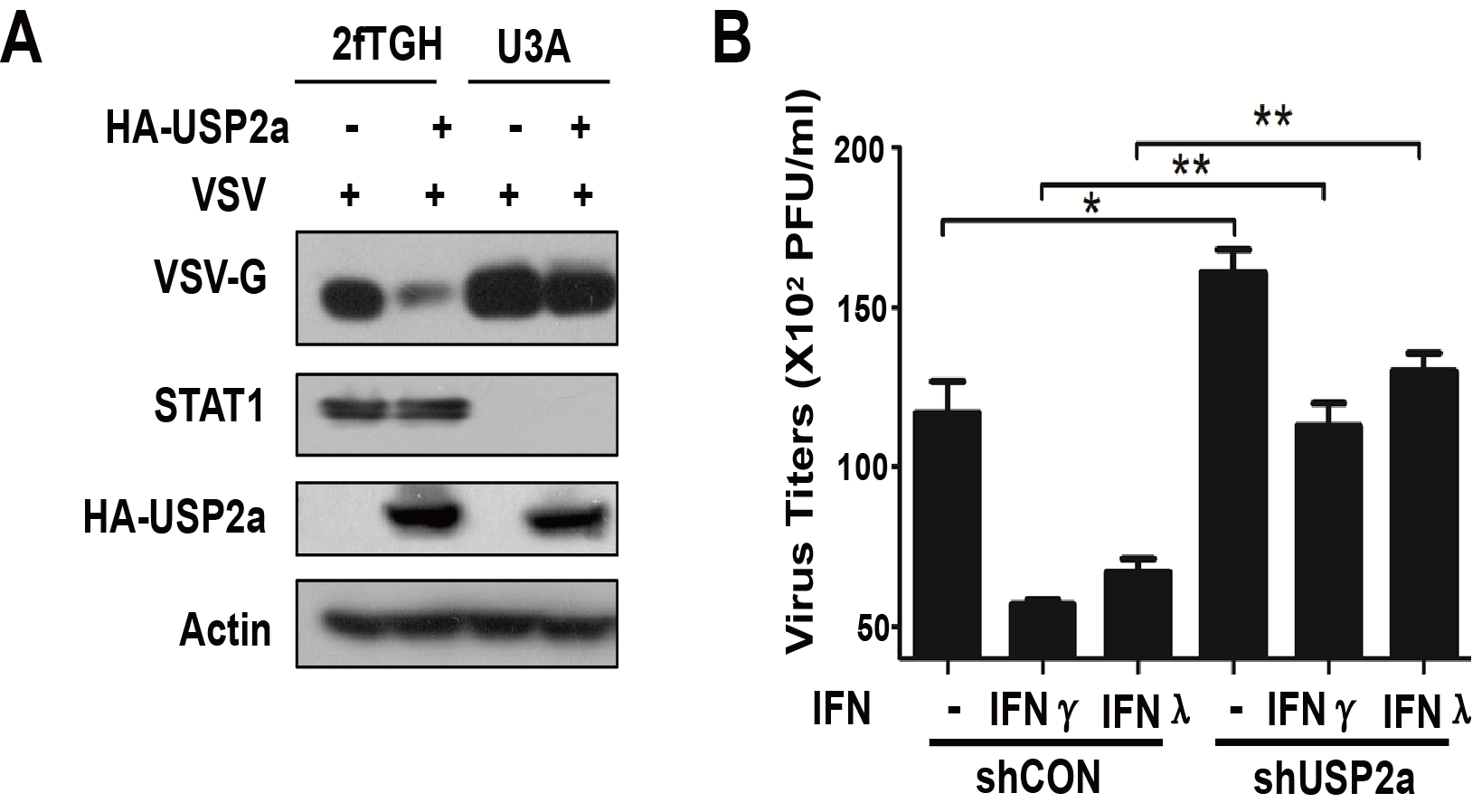

Supplement: S8 Fig — (A) 2fTGH and U3A cells transfected with or without HA-USP2a were infected with VSV (MOI = 1.0). After 20 hrs, cells were collected and immunoblotting was performed as indicated. (B) 293T cells transfected with shCON or shUSP2a were treated with IFNγ (100 IU/ml) or IFNλ (0.5 ng/ml) overnight, and then cells were challenged by VSV-GFP (MOI = 0.5). After 24 hrs, cell culture supernatant was collected, and a plaque assay was used for analysis of infectious viral titers. *p<0.05, **p<0.01, ***p<0.001. (TIF) [file ppat.1005764.s008.tif]
