# Supplementary material for: Deubiquitinase USP2a Sustains Interferons Antiviral Activity by Restricting Ubiquitination of Activated STAT1 in the Nucleus
Source: PLoS Pathog. 2016 Jul 19;12(7):e1005764. doi: 10.1371/journal.ppat.1005764 (PMC4951015; doi:10.1371/journal.ppat.1005764)
Supplement: S1 Table — (DOCX) [file ppat.1005764.s009.docx]

**S1 Table. List of ID numbers for genes and proteins**

| **Gene** | **ID** |  | **Protein** | **ID** |
| --- | --- | --- | --- | --- |
| *stat1* | GU211347.1 |  | STAT1 | ADA59516.1 |
| *usp2a* | NM_004205.4 |  | USP2a | NP_004196.4 |
| *tc-ptp* | BC016727.1 |  | TC-PTP | AAH16727.1 |
| *shp-2* | EU779708.1 |  | SHP-2 | ACE60556.1 |
| *kpna1* | NM_002264.3 |  | KPNA1 | NP_002255.3 |
| *jak1* | AB219242.1 |  | JAK1 | BAE02826.1 |
| *tyk2* | NM_003331.4 |  | Tyk2 | NP_003322.3 |
| *smurf1* | BC136804.1 |  | Smurf1 | AAI36805.1 |
| *usp13* | NM_003940.2 |  | USP13 | NP_003931.2 |
| *ifit1* | BC007091.1 |  | IFIT1 | AAH07091.1 |
| *isg15* | NM_005101.3 |  | ISG15 | NP_005092.1 |
| *isg54* | NM_001547.4 |  | ISG54 | NP_001538.4 |
| *mx1* | AF135187.1 |  | MX1 | AAD43063.1 |

These ID numbers for genes and proteins in this study are from NCBI database.
